# Aberrant activation of a miR-101–UBE2D1 axis contributes to the advanced progression and chemotherapy sensitivity in human hepatocellular carcinoma

**DOI:** 10.1038/s41420-024-02193-y

**Published:** 2024-10-01

**Authors:** Xiuli Mu, Yuchen Wei, Xin Fan, Rui Zhang, Wenjin Xi, Guoxu Zheng, An-gang Yang

**Affiliations:** 1 State Key Laboratory of Holistic Integrative Management of Gastrointestinal Cancers and Department of Immunology, Fourth Military Medical University, Xi’an, Shaanxi China; 2 State Key Laboratory of Holistic Integrative Management of Gastrointestinal Cancers and Department of Biochemistry and Molecular Biology, Fourth Military Medical University, Xi’an, Shaanxi China; 3 Department of Otolaryngology Head and Neck Surgery, Tangdu Hospital, Fourth Military Medical University, Xi’an, Shaanxi China

**Keywords:** Cancer, Biomarkers

## Abstract

Chemotherapeutic drugs, such as cisplatin (cis-dichlorodiamineplatinum [II], cDDP) and 5-fluorouracil (5Fu), are widely used in transarterial chemoembolization (TACE), which is a standard therapy for patients with hepatocellular carcinoma (HCC). Chemoresistance is a major cause of TACE treatment failure in HCC patients. Our previous studies have identified the expression levels of miR-101 responsive genes, such as EED, EZH2, STMN1 and JUNB, exhibit significant correlation with the occurrence and progression of HCC, while the role of miR-101 responsive gene signatures in the chemoresistance of HCC treatment remains unclear. In this study, we identified ubiquitin-coupled enzyme E2D1 (UBE2D1) as a crucial regulatory factor in the chemoresistance of HCC, which is a direct target of miR-101 and exhibits significant correlation with miR-101-responsive gene signatures. The bioinformatics analysis showed the expression of UBE2D1 was significantly increased in HCC tissues and was closely correlated with the poor prognosis. In addition, we analyzed the role of miR-101/UBE2D1 axis in regulating chemo-sensitive of HCC cells. Our results showed that miR-101 increases the DNA damage and apoptosis of HCC cells by inhibiting the expression of UBE2D1, which in turn increases the sensitivity of HCC cells to cDDP and 5Fu both in vitro and in vivo. Therefore, simultaneous assessment of miR-101 and UBE2D1 expression levels might provide an effective approach in preselecting HCC patients with survival benefit from TACE treatment. Moreover, further elucidation of the underlying molecular mechanisms of the miR-101/UBE2D1 axis could provide novel insight for targeted therapy of HCC.

## Introduction

Based on the most recent statistical data from 2020, primary liver cancer is identified as the third most common cause of cancer-related deaths worldwide, with hepatocellular carcinoma (HCC) representing the majority of cases at a rate of 75%–85% [1]. The predominant risk factors for HCC exhibits regional variation. In China, Hepatitis B virus (HBV) is a significant contributor to HCC morbidity, whereas in western countries, HCC incidence is primarily linked to hepatitis C virus (HCV) infection and alcohol consumption [2]. Early-stage HCC can be effectively managed with a variety of treatment modalities; however, a significant proportion of patients, ~70%–80%, are diagnosed at an unresectable advanced stage, resulting in poor survival rates [3, 4]. Transarterial chemoembolization (TACE) is considered a standard treatment option for patients with HCC, providing a viable alternative for individuals who are not candidates for surgical intervention or serving as an adjunctive therapy following surgery to mitigate the risk of recurrence [5, 6].

TACE initiates cellular apoptosis and ischemic necrosis within liver tumors by administering embolic agents and chemotherapeutic agents through arterial routes. Anthracyclines, platinum and fluorouracil are frequently employed chemotherapeutic agents in TACE procedures [5, 7]. Cisplatin (cis-dichlorodiamineplatinum [II], cDDP) and other platinum compounds demonstrate broad-spectrum efficacy and are widely used in the treatment of over 80% of malignancies [8]. The structural features of cDDP result in its ability to impede DNA replication and disrupt transcription, leading to the induction of DNA damage. The subsequent delayed repair of this damage frequently initiates the cellular apoptosis pathway [9, 10]. 5-Fluorouracil (5Fu) is commonly utilized in the treatment of diverse solid tumors through the inhibition of deoxythymidine synthase, thereby impacting intracellular DNA synthesis. Nevertheless, the potential for diminished efficacy, whether through inherent or acquired resistance, presents a significant obstacle to the optimal utilization of this chemotherapeutic agent [11]. Various mechanisms of drug resistance exist, including alterations in apoptosis-induced signaling proteins and the tumor microenvironment (TME), as well as the involvement of microRNAs (miRNAs) [12–14].

MiRNAs are a subset of small non-coding RNAs typically consisting of 20–25 nucleotides. Within eukaryotic organisms, miRNAs function by binding to the 3′ untranslated region (3′UTR) of target messenger RNAs (mRNAs) post-transcriptionally, thereby modulating a wide array of biological processes including but not limited to cell development, infection, immunity, carcinogenesis and chemoresistance [15, 16]. The impact of miRNAs on chemoresistance is associated with its ability to enhance drug efflux, interfere with cell cycle progression, and facilitate apoptosis [17]. Previous study has shown that miR-101 can be functioning as a tumor suppressor or a facilitator of cancer cell apoptosis via targeting crucial oncogenes and anti-oncogenes. Its efficacy in impeding the proliferation of various cancer types, including HCC, has been empirically validated [18]. In HCC, studies have shown that miR-101 inhibits tumorigenicity and participates in cellular life activities by targeting genes such as Mcl-1, EED, EZH2, and VEGF [18–22]. The specific mechanism by which miR-101 impacts DNA damage repair and drug resistance in HCC remains incompletely understood. As such, we hypothesized that miRNAs and their associated signaling pathways may play a role in the resistance of HCC to TACE.

The ubiquitination pathway is also a key factor in regulating cell proliferation, apoptosis and DNA damage repair [23]. Ubiquitination plays a critical role in mediating the cellular response to DNA double-strand breaks (DSBs), with delayed repair potentially resulting in genomic instability and tumorigenesis. The ubiquitin-conjugating enzyme E2D1 (UBE2D1), a member of the E2 family, is involved in various cancer-related signaling pathways through its ubiquitination modification mechanism [24]. Previous studies have shown that UBE2D1 can mediate the ubiquitination of tumor suppressor protein p53, KRAS, TNFα-NFκB [25–29] and other genes to participate in important pathways of carcinogenesis. UBE2D1 is believed to play a role in DNA damage repair, with increased levels of IL-6 in the serum of HCC patients triggering the DNA damage response and genomic instability, resulting in the repeated amplification of UBE2D1 gene copies in the genome [30]. Additionally, a previous study has shown that UBE2D1 aggregates at DSB sites and works in conjunction with RNF138 to promote CtIP ubiquitination and accumulation, thereby enhancing the efficiency of DNA repair [24]. Therefore, UBE2D1 may serve as a pivotal regulator of chemoresistance in HCC.

This study has established a significant correlation between UBE2D1 and miR-101-responsive gene signatures, demonstrating the role of the miR-101/UBE2D1 axis in mediating resistance to cDDP and 5Fu by enhancing DNA damage repair mechanisms. Our initial exploration of the oncogenic potential of UBE2D1 in HCC revealed that UBE2D1 is directly regulated by miR-101, influencing DNA damage repair and apoptosis processes, ultimately leading to resistance to both cDDP and 5Fu in HCC cells. In conclusion, targeting UBE2D1 combined with miR-101 may prove to be an effective strategy for overcoming multidrug resistance during chemotherapy for HCC.

## Results

### Screening and identification of UBE2D1 as a potential candidate is closely associated with miR-101-responsive gene signatures

**Fig. 1:**
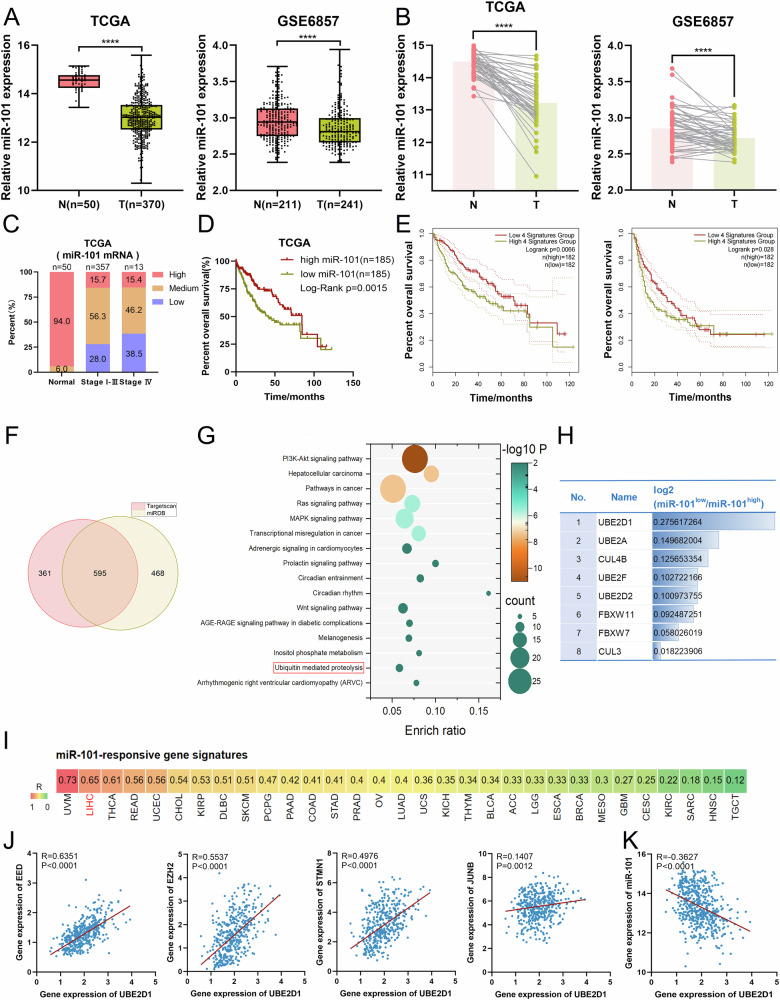
Expression of miR-101 in HCC patients and screening and identification of its downstream target gene UBE2D1. **A** Expression analysis of miR-101 in normal hepatic tissues (N) and LIHC tumor tissues (T) was conducted using data from the TCGA database and the GSE6857 dataset. **B** The differential expression of miR-101 between normal and paired LIHC tissues was analyzed using data from the TCGA database and the GSE6857 dataset. **C** Distribution of high, medium and low expression of miR-101 in HCC patients with different malignant degrees based on TCGA data. **D** The correlation between miR-101 expression levels and the overall survival of HCC patients was assessed using the Log-rank test. **E** The correlation between miR-101-responsive gene signatures (EED, EZH2, STMN1, JUNB) expression levels and the overall survival of HCC patients was derived from GEPIA. **F** Overlap of the predicted downstream targets of miR-101 from TargetScan and miRDB prediction databases. **G** The overlapping 595 predicted genes were subjected to KEGG analysis and enriched to the ubiquitin-mediated proteolysis pathway. The pathways were categorized based on the −log10 *P* value interval. **H** Fold-change differences with miR-101^low^/miR-101^high^ based on TCGA data, UBE2D1 showed the most pronounced alteration in expression. **I** Correlation analysis of UBE2D1 and miR-101-responsive gene signatures in different tumors according to GEPIA. **J** A positive correlation was observed between the expression levels of UBE2D1 and EED, EZH2, STMN1, JUNB in LIHC tissues based on TCGA data. **K** A negative correlation was observed between the expression levels of UBE2D1 and miR-101 in LIHC tissues based on TCGA data. Statistical significance was determined by Student’s *t*-test. ****p < 0.0001.

MiR-101 is thought to exhibit tumor suppressive properties in multiple types of malignancies. To verify its role in HCC, we analyzed the expression of miR-101 using clinical data obtained from the TCGA-LIHC cohort and the GSE6857 dataset. Both datasets consistently showed that the expression of miR-101 in LIHC tissues was dramatically reduced when compared with normal tissues (Fig. 1A). The TCGA dataset contained 49 pairs of LIHC tissues and matched normal samples, and GSE6857 contained 48 paired samples, and the expression of miR-101 in these tumor tissues showed an overall downward trend relative to corresponding normal tissues (Fig. 1B). Moreover, there was a significant correlation observed between miR-101 expression levels, WHO grade, and survival outcomes in HCC patients. A higher grade of malignancy was found to be associated with a greater prevalence of low miR-101 expression, while the converse was true for lower degrees of malignancy (Fig. 1C). Patients exhibiting low levels of miR-101 expression experienced a significantly reduced survival duration in comparison to those with elevated levels (Fig. 1D). The above findings confirm the independent prognostic significance of miR-101 in HCC. The expression of EED, EZH2, STMN1 and JUNB [31] was closely associated with miR-101 and GEPIA analysis revealed that the elevated expression of these miR-101-responsive gene signatures was correlated with poor prognosis in HCC patients (Fig. 1E). Further research is needed to explore the potential clinical significance of molecules with characteristics similar to miR-101-responsive gene signatures in the diagnosis and prognosis of HCC patients.

Next, we utilized TargetScan and miRDB to identify potential downstream target genes regulated by miR-101 (Fig. 1F). A total of 595 novel underlying molecular targets of miR-101 in the intersection were used for KEGG analysis. The ubiquitin-mediated proteolysis pathway exhibited enrichment in eight ubiquitination-related molecules, specifically UBE2D1, UBE2A, CUL4B, UBE2F, UBE2D2, FBXW11, FBXW7 and CUL3 (Fig. 1G, H). Based on the mean cutoff value of miR-101 expression, we compared the expression changes of these ubiquitinating factors between HCC patients with high miR-101 levels (named as miR-101^high^) and low miR-101 levels (named as miR-101^low^). The log2 ratio of miR-101^low^ to miR-101^high^ was utilized to determine the fold change in the expression levels of miR-101 between groups exhibiting high and low miR-101 expression. UBE2D1 demonstrated the most pronounced alteration in expression (Fig. 1H). Following correlation analysis, the relationship between UBE2D1 and miR-101-responsive gene signatures in LIHC was found to be the second highest among various types of tumors (Fig. 1I). The evidence presented also indicated a positive correlation between the expression levels of UBE2D1 and miR-101-responsive gene signatures (Fig. 1J). However, the expression levels of miR-101 was negatively correlated with UBE2D1 in LIHC tissues (Fig. 1K). Therefore, it is worth exploring whether UBE2D1 is regulated by miR-101 and involved in HCC progression.

### Abnormally high expression of UBE2D1 is closely associated with poor prognosis of HCC patients in multiple clinical cohorts

**Fig. 2:**
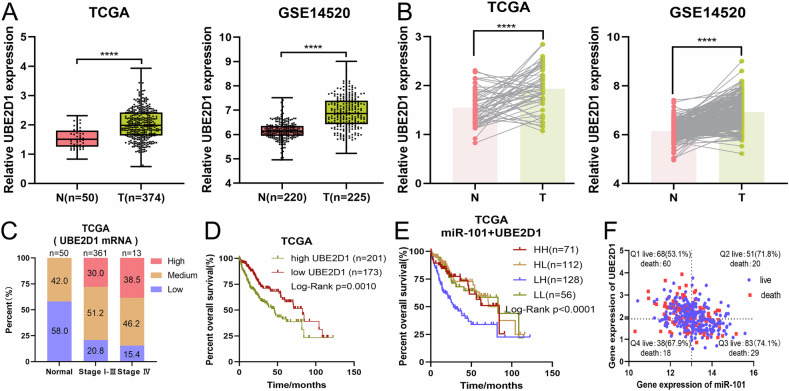
Abnormally high expression of UBE2D1 is closely associated with poor prognosis of HCC patients. **A** Analysis of UBE2D1 expression in normal hepatic tissues (N) and LIHC tumor tissues (T) was conducted using data from the TCGA database and the GSE14520 dataset. **B** The differential expression of UBE2D1 between normal and paired LIHC tissues was analyzed by utilizing data from both the TCGA database and the GSE14520 dataset. **C** Distribution of high, medium and low expression of UBE2D1 in HCC patients with varying degrees of malignancy based on TCGA data. **D** Overall survival rate of HCC patients from the TCGA database was analyzed based on UBE2D1 mRNA levels using the Log-rank test. **E** Survival curve of 367 LIHC patients enrolled in TCGA database. Patients were classified into four groups based on the expression levels of miR-101 and UBE2D1 mRNA in their tumors. **F** The expression patterns of miR-101 and UBE2D1 mRNA in 367 LIHC samples and the statistical analysis of patient survival outcomes. Statistical significance was determined by Student’s *t*-test. ****p < 0.0001.

To investigate the potential oncogenic role of elevated UBE2D1 expression in HCC, we utilized the TCGA-LIHC cohort and the GSE14520 dataset to merge clinical data with genomic information on UBE2D1 for subsequent analysis. Our findings revealed a statistically significant increase in UBE2D1 expression levels in LIHC tissues compared to adjacent tissues, indicating a consistent upward trend relative to paired normal tissues (Fig. 2A, B). Furthermore, there was a significant correlation between UBE2D1 expression and the WHO grade of patients with HCC, with a greater prevalence of elevated UBE2D1 expression in cases of higher malignancy, and conversely, a lower incidence in cases of lower malignancy (Fig. 2C). In order to investigate the potential correlation between the expression levels of UBE2D1 and the clinical prognosis of patients with HCC, survival analysis was conducted. The results indicated that elevated levels of UBE2D1 expression in tumor tissues were significantly linked to decreased overall survival rates in HCC patients (Fig. 2D). In conclusion, abnormally high expression of UBE2D1 can be used as a poor prognostic factor for the overall survival in HCC patients.

To facilitate further data analysis, we employed appropriate critical values to divide LIHC tumor samples into high and low miR-101 expression groups, as well as high and low UBE2D1 expression groups, and then combined into four groups: miR-101^high^UBE2D1^high^, miR-101^high^UBE2D1^low^, miR-101^low^UBE2D1^high^, miR-101^low^UBE2D1^low^ for survival comparison purposes. As hypothesized, patients classified within the miR-101^high^UBE2D1^low^ group demonstrated the most favorable prognosis, whereas those categorized in the miR-101^low^UBE2D1^high^ group exhibited the poorest prognosis (Fig. 2E). Divided by four quadrants, the highest survival rate was observed in the miR-101^high^UBE2D1^low^ group whereas the lowest survival rate was found in the miR-101^low^UBE2D1^high^ group (Fig. 2F). The observed expression patterns of miR-101 and UBE2D1 in HCC patients exhibit a strong correlation with overall survival rates, thereby offering valuable insights for prognostic prediction within this specific patient cohort.

### Knockdown of UBE2D1 enhances sensitization of HCC cells to chemotherapeutic drug-induced cell death

**Fig. 3:**
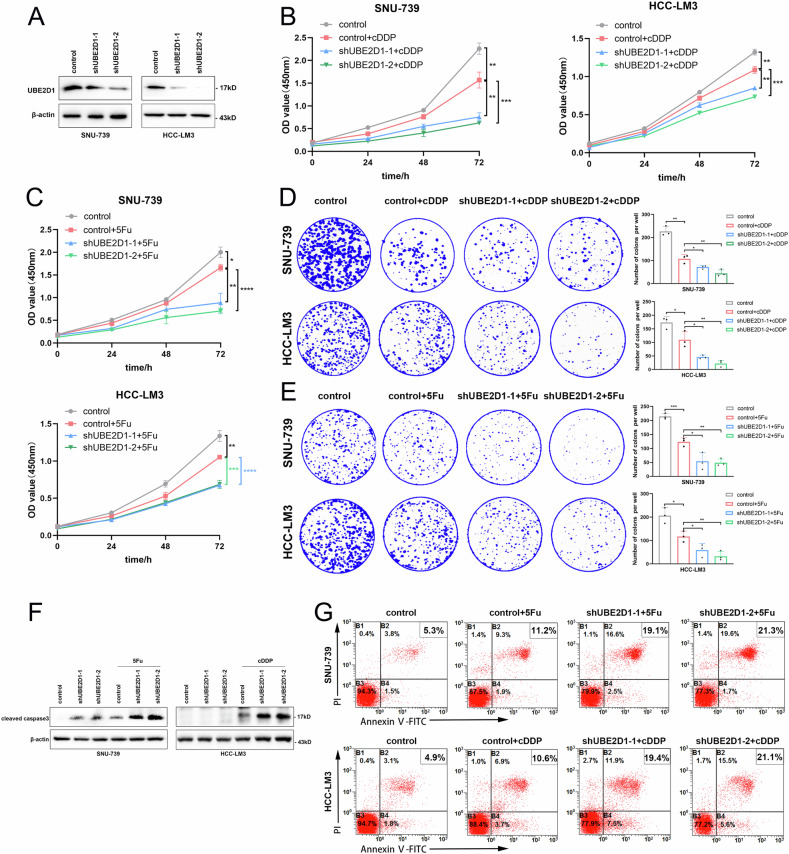
Knockdown of UBE2D1 enhances sensitization of HCC cells to chemotherapeutic drug-induced cell death. **A** Protein levels identification of shRNA-mediated UBE2D1 knockdown effect in SNU-739 and HCC-LM3 cells. **B**, **C** Cell viability was analyzed in stable UBE2D1-knockdown cell lines following treatment of 0.5 μM cDDP for 0, 24, 48 and 72 h respectively and treatment of 0.5 μM 5Fu for 0, 24, 48 and 72 h respectively by CCK8 assay. **D**, **E** Cell proliferation ability detection of stable UBE2D1-knockdown cell lines after treatment with 0.5 μM cDDP or 0.5 μM 5Fu by plate clone formation assay. **F** A western blotting analysis of cleaved caspase 3 protein levels in UBE2D1-knockdown cells with or without the cDDP (50 μM, 24 h) or 5Fu (50 μM, 48 h) treatment. **G** Apoptosis rate detection of UBE2D1-knockdown cells after treatment with cDDP or 5Fu (5 μM, 24 h) by flow cytometry. All experiments were performed three times. Statistical significance was determined by Student’s *t*-test. ****p < 0.0001, ***p < 0.001, **p < 0.01, *p < 0.05.

In order to investigate the function of UBE2D1 in various HCC cell lines, we established stable UBE2D1-knockdown cell lines in SNU-739 and HCC-LM3 cells through lentiviral transduction with two UBE2D1-specific shRNAs, while control cells were transduced with an empty pLKO.1 vector lentivirus. The results indicated that specific shRNAs were able to effectively knockdown endogenous UBE2D1 protein in SNU-739 and HCC-LM3 cell lines (Fig. 3A). The cell proliferation was analyzed by CCK8 assay, revealing a proliferation inhibitory effect on SNU-739 and HCC-LM3 cells via reduction of UBE2D1 expression (Fig. S1A). To evaluate the impact of UBE2D1 on chemosensitivity in HCC, we employed cDDP and 5Fu at the minimal concentrations that effectively inhibited cell proliferation to treat stable transfected cell lines (Fig. S1B). In both SNU-739 and HCC-LM3 cells, the knockdown of UBE2D1 resulted in a notable enhancement in the susceptibility of the cells to chemotherapeutic agents, as evidenced by reduced rates of cell proliferation and colony formation (Fig. 3B–E). In addition, knockdown of UBE2D1 resulted in a notable increase of cleaved caspase 3 protein, a reliable marker of apoptosis (Fig. 3F). Similarly, flow cytometry analysis showed that UBE2D1 knockdown significantly increased the apoptosis rate after treatment with cDDP or 5Fu (Fig. 3G).

### High expression of UBE2D1 attenuates sensitization of HCC cells to chemotherapeutic drug-induced cell death

**Fig. 4:**
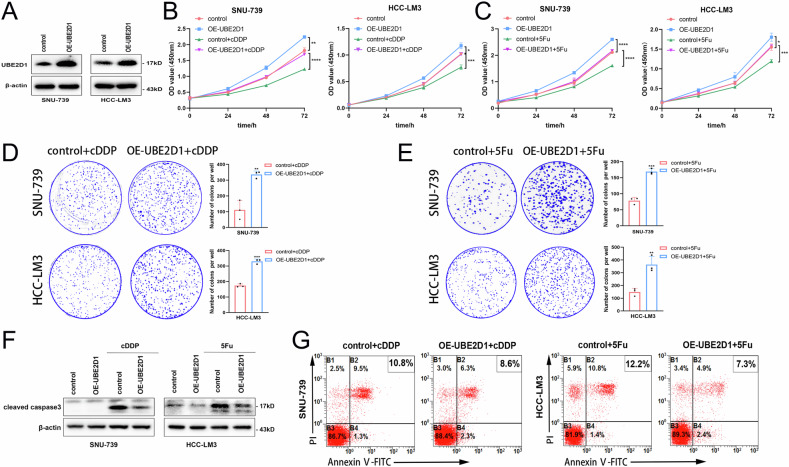
High expression of UBE2D1 attenuates sensitization of HCC cells to chemotherapeutic drug-induced cell death. **A** Protein levels identification of UBE2D1 overexpression effect in SNU-739 and HCC-LM3 cells. **B**, **C** Cell viability was assessed in stable UBE2D1-overexpression cells following treatment of 0.5 μM cDDP for 0, 24, 48, and 72 h respectively and treatment of 0.5 μM 5Fu for 0, 24, 48, 72 h respectively by CCK8 assay. **D**, **E** The cell proliferation ability of stable UBE2D1-overexpression cells was assessed after treatment with 0.5 μM cDDP or 0.5 μM 5Fu using plate clone formation assay. **F** A western blotting analysis of cleaved caspase 3 protein levels in stable UBE2D1-overexpression cells with or without the cDDP (50 μM, 24 h) or 5Fu (50 μM, 48 h) treatment. **G** Apoptosis rate of stable UBE2D1-overexpression cells was assessed by flow cytometry after treatment with cDDP or 5Fu (5 μM, 24 h). All experiments were performed three times. Statistical significance was determined by Student’s *t*-test. ****p < 0.0001, ***p < 0.001, **p < 0.01, *p < 0.05.

To investigate the impact of UBE2D1 overexpression on chemotherapy resistance, SNU-739 and HCC-LM3 cells were infected with lentivirus carrying either control or UBE2D1-overexpressing constructs. Following western blotting analysis, our findings indicated that HCC cells exhibited successful upregulation of UBE2D1 (Fig. 4A). The efficacy of chemotherapy agents was markedly reduced following the upregulation of UBE2D1 in SNU-739 and HCC-LM3 cells, as indicated by enhanced cellular proliferation and colony formation (Fig. 4B–E). In addition, the overexpression of UBE2D1 resulted in a significant reduction in caspase 3 cleavage and apoptosis rate after treatment with cDDP or 5Fu (Fig. 4F, G).

### UBE2D1 attenuates DNA damage induced by chemotherapy drugs in HCC cells

**Fig. 5:**
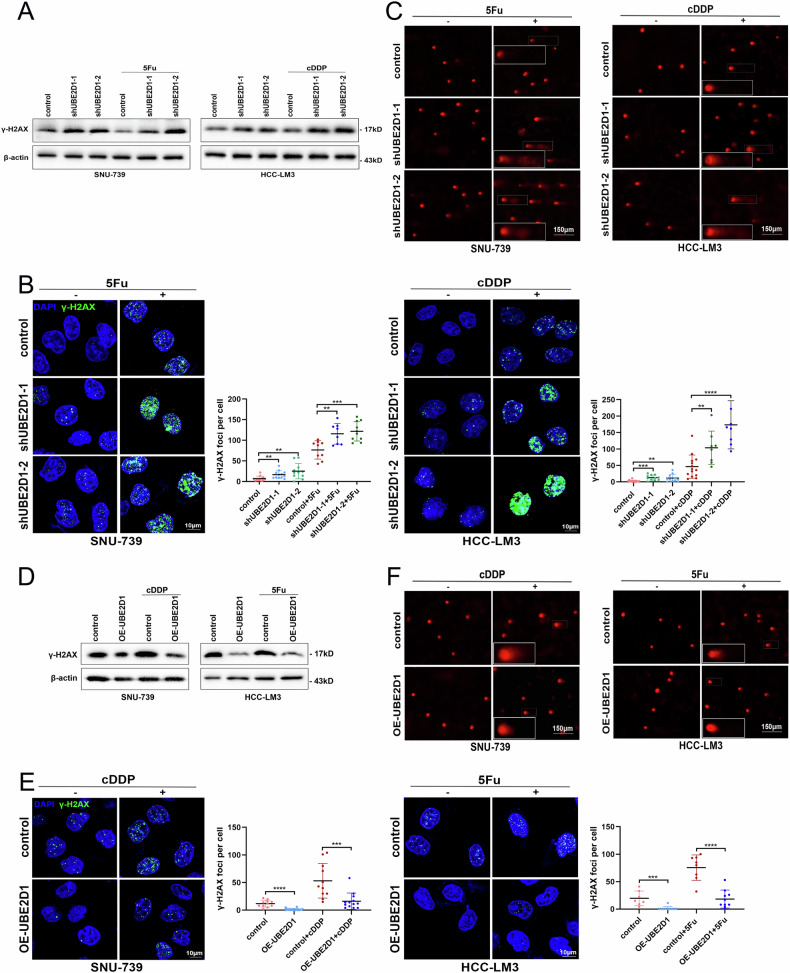
UBE2D1 attenuates DNA damage induced by chemotherapy drugs in HCC cells. **A** Western blotting analysis of γ-H2AX protein levels in HCC cells following stable knockdown of UBE2D1, with or without treatment of cDDP or 5Fu (10 μM, 2 h). **B** Representative images and quantification of γ-H2AX foci in stable UBE2D1-knockdown cells following treatment with or without cDDP or 5Fu (10 μM, 2 h). γ-H2AX accumulates at DNA double-strand break sites (scale bar = 10 μm). The statistical scatter points represent the number of γ-H2AX foci in each cell, which were counted by Image J software. **C** The comet assay was employed to evaluate the extent of DNA damage in stable UBE2D1-knockdown cells treated with or without cDDP or 5Fu (10 μM, 12 h). The head of the comet as a spherical mass of undamaged DNA, while the damaged DNA (DNA loops around strand breaks) emanates from the head in the form of a tail (scale bar = 150 μm). **D** Western blotting analysis of γ-H2AX protein levels in HCC cells following stable overexpression of UBE2D1, with or without the cDDP or 5Fu treatment (10 μM, 2 h). **E** Representative images and quantification of γ-H2AX foci in stable UBE2D1-overexpression cells following treatment with or without cDDP or 5Fu (10 μM, 2 h) (scale bar = 10 μm). **F** Comet assay was performed on stable UBE2D1-overexpression cells, with or without treatment of cDDP or 5Fu (10 μM, 12 h) (scale bar = 150 μm). All experiments were performed three times. Statistical significance was determined by Student’s *t*-test. ****p < 0.0001, ***p < 0.001, **p < 0.01.

To investigate whether UBE2D1 induces chemoresistance by attenuating DNA damage and inhibiting cell apoptosis, we performed DNA damage assays. The formation of nuclear γ-H2AX foci at DNA damage sites is the earliest marker of DNA damage occurs [32]. In order to evaluate this sensing phenomenon, western blotting and immunofluorescence staining were performed, and the results revealed that reduced UBE2D1 significantly increased the occurrence of γ-H2AX after treatment with cDDP and 5Fu (Fig. 5A, B). The comet assay, employed for evaluating the levels of DNA damage, revealed a correlation between decreased levels of UBE2D1 and an increase in DNA damage, with a notable accumulation of damage observed when treated with cDDP and 5Fu (Fig. 5C). In contrast, the overexpression of UBE2D1 led to a decrease in DNA damage, consequently diminishing the susceptibility of cells to chemotherapeutic agents. This was supported by the decreased presence of γ-H2AX and shortened tailing induced by DNA damage (Fig. 5D–F).

### UBE2D1 is identified as a de novo target of miR-101 in HCC cells

**Fig. 6:**
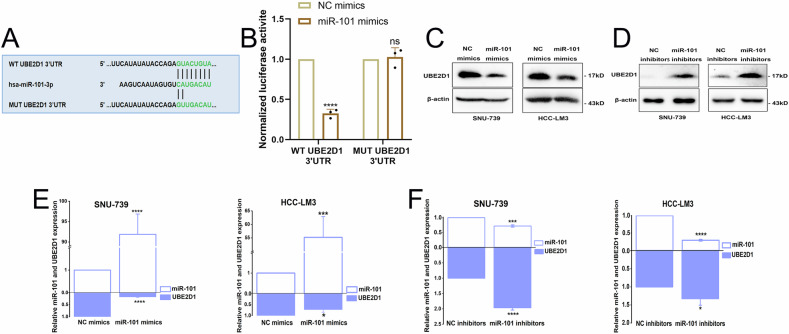
UBE2D1 is identified as a de novo target of miR-101 in HCC cells. **A** The predicted binding sites between the wild-type (WT) and mutant (MUT) 3ʹUTR of UBE2D1 and miR-101. **B** WT UBE2D1 3ʹUTR and MUT UBE2D1 3ʹUTR firefly luciferase activities were measured in HEK293T cells transfected with NC mimics or miR-101 mimics. **C**, **D** Western blotting analysis of UBE2D1 expression in SNU-739 and HCC-LM3 cells transfected with miR-101 mimics or miR-101 inhibitors. **E**, **F** The miR-101 and UBE2D1 transcript levels were quantified by qRT-PCR in cells transfected with miR-101 mimics or miR-101 inhibitors. Graph represents miR-101 and UBE2D1 mRNA levels, expressed as fold changes relative to the control. All experiments were performed three times. Statistical significance was determined by Student’s *t*-test. ****p < 0.0001, ***p < 0.001, *p < 0.05, ns nonsignificant.

As shown the predicted binding site between the 3ʹUTR of UBE2D1 and miR-101 according to the TargetScan online tool (Fig. 6A). In order to investigate the potential targeting of UBE2D1 by miR-101, plasmids containing either matching or mutant 3ʹUTR sequences of UBE2D1 were generated and transfected into HEK293 cells along with miR-101 mimics. Subsequent luciferase reporter assays revealed a significant reduction in relative luciferase activity of the reporter gene containing the wild-type UBE2D1-3ʹUTR in SNU-739 and HCC-LM3 cells upon transfection with miR-101 mimics. Conversely, no significant change in relative luciferase activity was observed in cells transfected with the mutant UBE2D1-3ʹUTR reporter gene (Fig. 6B). Moreover, the results of western blotting and qRT-PCR analysis revealed that miR-101 mimics could significantly suppress UBE2D1 protein and mRNA expression in SNU-739 and HCC-LM3 cells (Fig. 6C, E). In cells transfected with miR-101 inhibitors, the opposite result was observed (Fig. 6D, F).

### The miR-101–UBE2D1 axis enhances HCC chemosensitivity by increasing DNA damage

**Fig. 7:**
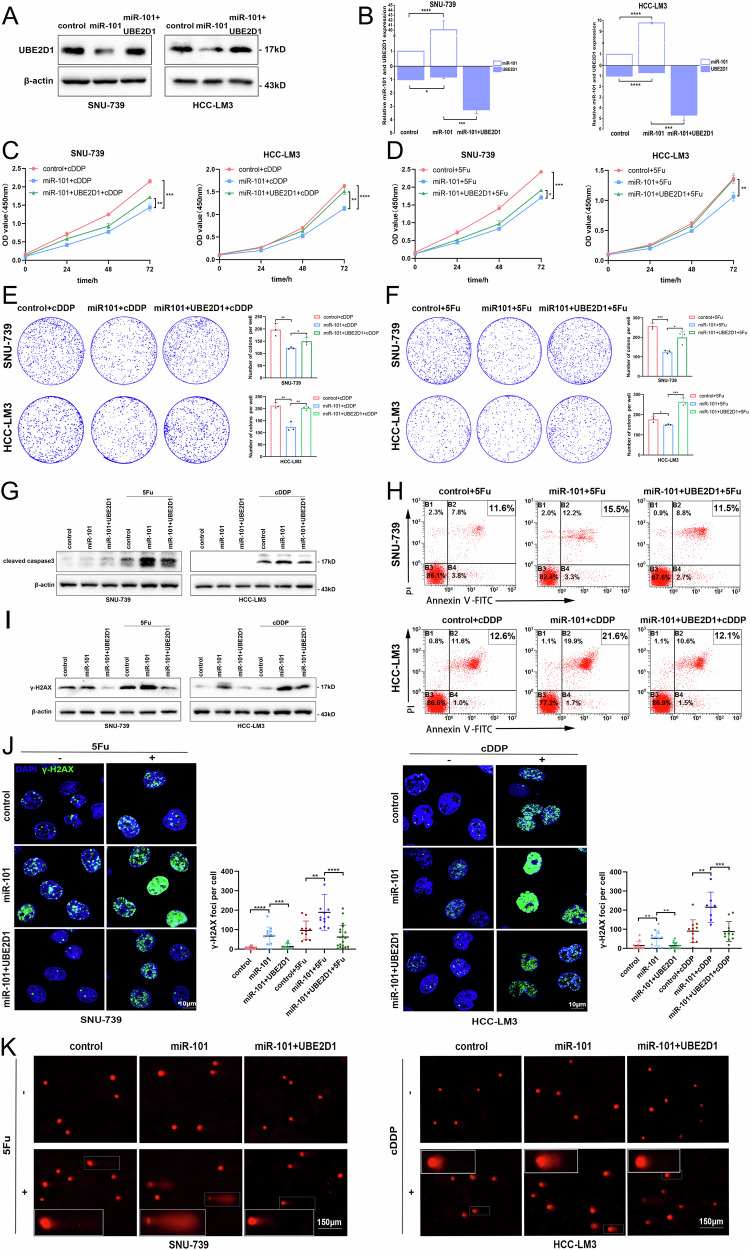
The MiR-101–UBE2D1 axis enhances HCC chemosensitivity by increasing DNA damage. **A** Western blotting analysis of UBE2D1 expression changes in SNU-739 and HCC-LM3 cells following stable transfection of miR-101 and rescue expression of UBE2D1 on this basis. **B** The miR-101 and UBE2D1 transcript levels were quantified by qRT-PCR in miR-101 overexpression cells and rescued cells. **C**, **D** Cell proliferation of HCC cells was analyzed in the rescue experiments following treatment of 0.5 μM cDDP or 0.5 μM 5Fu for 0, 24, 48 and 72 h respectively by CCK8 assay. **E**, **F** The cell proliferation of HCC cells was analyzed in the rescue experiments after treatment with 0.5 μM cDDP or 0.5 μM 5Fu using plate clone formation assay. **G** Cleaved caspase 3 expression levels were analyzed using western blotting in the rescue experiments performed on SNU-739 and HCC-LM3 cells, with or without administration of cDDP (50 μM, 24 h) or 5Fu (50 μM, 48 h). **H** Cell apoptosis rate was detected by flow cytometry in the rescue experiments with the cDDP or 5Fu treatment (5 μM, 24 h). **I**, **J** Detection of γ-H2AX DNA damage foci in the rescue experiments of SNU-739 and HCC-LM3 cells by western blotting and immunofluorescent staining (scale bar = 10 μm). The statistical scatter points represent the quantification of γ-H2AX foci per cell. **K** Comet assay was performed on the rescue experiments with or without administration of cDDP or 5Fu (10 μM, 12 h) (scale bar = 150 μm). All experiments were performed three times. Statistical significance was determined by Student’s *t*-test. ****p < 0.0001, ***p < 0.001, **p < 0.01, *p < 0.05.

To explore the functional significance of miR-101 in cDDP and 5Fu resistance in HCC, we constructed SNU-739 and HCC-LM3 cells that stably overexpressed miR-101 and identified the UBE2D1 expression changes by western blotting and qRT-PCR analysis (Fig. 7A, B). the results of CCK8 assays and plate clone formation assays indicated that overexpression of miR-101 could inhibit the proliferation of SNU-739 and HCC-LM3 cells, while the rescue expression of UBE2D1 reversed the inhibition effect induced by miR-101 after treatment with cDDP and 5Fu (Fig. 7C–F).

Moreover, miR-101 overexpression combined with cDDP or 5Fu treatment considerably potentiate the expression of cleaved caspase 3 protein in SNU-739 and HCC-LM3 cells. In the rescue experiments, the rescue expression of UBE2D1 in miR-101 overexpression cells attenuated the expression of cleaved caspase 3 protein (Fig. 7G). Similarly, apoptosis rates were assessed by flow cytometry, which showed the same trends (Fig. 7H). The formation of γ-H2AX foci was detected by western blotting and immunofluorescence staining (Fig. 7I, J), as well as the comet assay (Fig. 7K) all showed that miR-101 enhanced chemotherapy-induced DNA damage via regulation of UBE2D1. These results suggest that the downregulation of UBE2D1 by miR-101 is able to sensitize HCC cells to chemotherapeutic drug-induced apoptosis.

### The miR-101–UBE2D1 axis regulates tumor growth and chemoresistance in vivo

**Fig. 8:**
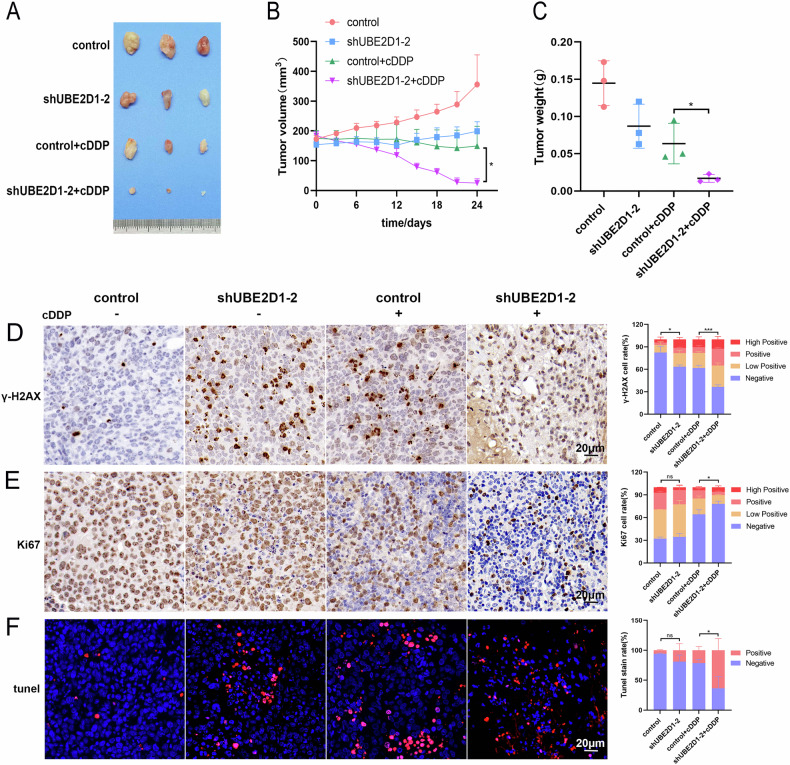
The MiR-101–UBE2D1 axis regulates tumor growth and chemoresistance in vivo. **A** Representative tumor images in nude mice after the injection of SNU-739 cells that stably expressing UBE2D1-shRNA and control scramble shRNA, with or without the cDDP treatment (2 mg/kg/3 days). **B**, **C** Growth curves of xenograft tumors in nude mice and quantification of tumor weight. The initiation time of drug administration is recorded as 0 d. **D–F** Representative images of γ-H2AX, Ki67 and tunel staining in tumor samples. On the right is the result of statistical analysis on positive signals using Image J Profiler and GraphPad Prism (scale bar = 20 μm). All experiments were performed three times. Statistical significance was determined by Student’s *t*-test. ***p < 0.001, *p < 0.05, ns nonsignificant.

The xenograft tumors of HCC were established to further investigate the function of UBE2D1 in vivo and its impact on the development of chemoresistance. UBE2D1 stable-knockdown cells of SNU-739 mixed with matrigel were injected subcutaneously into nude mice (male mice aged 5 weeks old; Beijing Vital River Laboratory Animal Technology Co., Ltd., China). When the tumor volume reached ~150 mm^3^, cDDP treatment (2 mg/kg/3 days) was initiated. As shown, tumor volume and tumor weight were significantly reduced in both the sh-UBE2D1 group and control + cDDP group compared to the control group. Notably, the tumor volume and weight of the shUBE2D1 + cDDP group were the lowest among all groups (Fig. 8A–C), indicating that UBE2D1 knockdown enhanced the inhibitory effect of cDDP on tumor growth. We further detected the expression levels of γ-H2AX and Ki67 in the tumors using IHC staining. The results showed that knockdown of UBE2D1 significantly enhanced DNA damage and inhibited cell proliferation activity, as indicated by the percentage of γ-H2AX and Ki67-stained positive cells compared with the control group (Fig. 8D, E). Tunel staining showed that UBE2D1 inhibition significantly increased chemotherapy-induced apoptosis (Fig. 8F).

## Discussion

TACE chemotherapy has been suggested as an initial treatment option for both primary and recurrent cases of liver cancer, with the potential to enhance patient survival outcomes to differing extents [33]. The methodology employed not only integrates the use of chemotherapy agents like cDDP and 5Fu for the eradication of tumors, but also initiates tumor vascular embolization and subsequent necrosis in order to impede or postpone tumor progression. Nevertheless, the persistence of drug resistance-induced tumor relapse and metastasis poses significant challenges to the prognosis of patients with liver cancer, with therapeutic shortcomings frequently stemming from a myriad of genetic and epigenetic modifications, as well as subsequent disruption of crucial genes and protein networks governing the fate of tumor cells [34, 35]. A thorough understanding of the tumor biological pathways underlying liver cancer, combined with the identification of genes responsive to chemotherapy agents to enhance drug sensitivity, will facilitate the implementation of precision medicine for patients and improve therapeutic outcomes in liver cancer. However, a limitation of our research is the exclusion of the TACE mouse model, owing to the challenges associated with its establishment.

MiRNA comprises only about 1% of the total genes in the human genome, yet it plays a regulatory role in ~60% of protein-coding genes, demonstrating significant functional potency [36]. To date, over 50% of human miRNAs have been found associated with carcinogenesis, suggesting that abnormal miRNA expression profiles could serve as a promising indicator of malignancy [11]. Among these, miR-101 is acknowledged as a tumor suppressor miRNA in a range of malignant tumors, as it demonstrates the capacity to modulate multiple oncogenes and play a role in the modulation of drug resistance across various cancer types [37–41]. In the context of liver cancer progression, a significant downregulation of miR-101 expression was observed in LIHC tissues relative to adjacent tissues, resulting in dysregulation of tumor suppressor pathways and correlation with disease stage. Deletion of miR-101 has been implicated in the acceleration of liver cancer development. Various external and internal factors, including atmospheric particles, viruses and pro-inflammatory cytokines, may contribute to alterations in miR-101 levels [42]. Therefore, our study sought to investigate the potential biological relevance of miR-101 in tumorigenesis, building upon prior research conducted by our group that established miR-101’s ability to suppress malignant characteristics in HCC cells by concurrently targeting oncogenes such as STMN1 (proliferation), JUNB (angiogenesis) and CXCR7 (migration/invasion) [31]. However, the precise mechanisms by which miR-101 modulates chemoresistance in HCC remain to be elucidated.

First, bioinformatics predictions were generated using TargetScan and miRDB online tools, followed by validation through molecular biology experiments. Our findings indicated that the ubiquitination factor UBE2D1 is a direct target gene negatively modulated by miR-101 at both the transcriptional and post-transcriptional levels in SNU-739 and HCC-LM3 cells. Importantly, a significant association was observed between UBE2D1 and miR-101-responsive gene signatures. Overexpression of UBE2D1 has been shown to be associated with poor prognosis in several human cancers, such as breast cancer, liver cancer, bladder cancer and lung adenocarcinoma [43–46]. These findings are consistent with the LIHC sample data information from the TCGA database. In addition, UBE2D1 had also been found to be overexpressed in precancerous lesions, and it was only upregulated in cancer cells rather than hepatitis cells, suggesting its potential as an early-stage HCC marker [30]. Subsequently, we investigated the impact of miR-101 and UBE2D1 on the proliferation of HCC cells following chemotherapy through a series of gain and loss of function experiments conducted both in vitro and in vivo. Silencing UBE2D1 using shRNA was found to enhance the sensitivity of HCC cells to cDDP and 5Fu, as evidenced by a significant reduction in cell viability and an increase in apoptosis. To further elucidate the role of UBE2D1 in miR-101-mediated chemosensitivity, we reintroduced UBE2D1 into miR-101-overexpressing cells and treated them with chemotherapeutic agents. The upregulation of miR-101 resulted in a significant inhibition of tumor cell proliferation and colony formation in vitro. Importantly, the inhibitory effects of miR-101 were found to be reversed by UBE2D1.

DNA damage is a crucial anti-cancer mechanism of action for most chemotherapy drugs. For instance, cDDP and 5Fu are commonly employed in TACE therapy, which can directly or indirectly induce DNA damage, induce apoptosis, and inhibit cell proliferation [5, 47]. DNA damage was identified through the detection of γ-H2AX using western blotting and immunofluorescence staining, which revealed the products formed following the occurrence of DNA damage. Additionally, based on the alteration of the physicochemical properties of damaged DNA, we assessed the extent of DNA damage with a comet experiment [48]. The results showed that downregulation of UBE2D1 and overexpression of miR-101 resulted in an increase in the population of HCC cells displaying DNA damage, as evidenced by the presence of distinct nuclear foci of γ-H2AX and migration of damaged DNA. This effect was consistently observed in two HCC cell lines, SNU-739 and HCC-LM3. In conclusion, miR-101 has been identified as a promoter of DNA damage by targeting and binding ubiquitinated proteins, which subsequently activates the ubiquitination pathway and modulates the sensitivity of HCC cells to cDDP and 5-fluorouracil treatments. However, the involvement of UBE2D1 in DNA damage repair has been previously documented. This suggests the potential existence of additional, yet undiscovered mechanisms that merit further investigation, particularly those elucidating the intricate processes of DNA damage repair.

MiRNA has long been advocated as a biomarker for early diagnosis and therapeutic efficacy assessment according to Chinese guidelines [49–51]. The miRNA panel consisting of miR-122, miR-192, miR-21, miR-223, miR-26a, miR-27a and miR-801 has high accuracy in diagnosing early HBV-associated HCC, with a 30% increase in sensitivity compared to AFP levels alone [51]. The miR-101/UBE2D1 signaling axis plays a crucial role in promoting genomic alterations in oncogenes during HCC carcinogenesis. Simultaneous evaluation of miR-101 and UBE2D1 could potentially serve as valuable biomarkers or therapeutic targets for predicting prognosis and disease severity in HCC. A comprehensive understanding of the molecular mechanisms involved in the regulation of the miR-101/UBE2D1 axis provides new perspectives for the diagnosis and targeted treatment of HCC, suggesting the potential benefit of combining UBE2D1 inhibition with conventional chemotherapy.

## Materials and methods

### Clinical data analysis and bioinformatics prediction

Curated datasets obtained from The Cancer Genome Atlas (TCGA) and Gene Expression Omnibus (GEO) were used for gene expression analysis, correlation analysis and overall survival (OS) analysis. For the prediction of canonical target genes of miR-101, TargetScan and miRDB prediction databases were used. In order to conduct further screening, KEGG pathway enrichment analysis was performed using online analysis tools DAVID and KOBAS, with both the screened *P* value and corrected *P* value being <0.01.

### Cell culture and drug treatment

For human HCC cell lines, SNU-739 and HCC-LM3 were cultured in RPMI-1640 medium (GIBCO BRL, Grand Island, NY, USA) supplemented with 10% FBS (GIBCO BRL) and 100 mg/ml penicillin–streptomycin at 37 °C in a 5% CO_2_ incubator. Similarly, the basal medium of HEK293T cells was changed to DMEM (GIBCO BRL) under the same culture conditions. All three cell lines were purchased from ATCC (Manassas, VA, USA). All cell lines were tested without mycoplasma contamination. For chemotherapeutic drugs, cDDP and 5Fu were purchased from Selleckchem catalog #S1166 and #S1209, respectively.

### Plasmid construction and lentiviral transduction

The short-hairpin sequences against human UBE2D1 (Table S1) were designed by Sigma-Aldrich. The synthesized primers were annealed and cloned into the pLKO.1-TRC vector (Addgene, Cambridge, USA; 10878) to obtain plasmids for stable knockdown of UBE2D1 (shUBE2D1). The UBE2D1 overexpression plasmid was constructed by cloning the open reading frame of the cDNA into the polyclonal sites of pCDH vector, and the primers used were shown in Table S2. A pLenti6.3/V5-DEST vector was used to construct a plasmid for rescuing UBE2D1. The pCDH-miR-101 plasmid was retained in our laboratory.

When HEK293T cells reached 60%–80% confluence, the target plasmid, packaging plasmid psPAX2 (Addgene, 12260) and envelope plasmid pMD2.G (Addgene, 12259) were mixed at a mass ratio of 4:3:1 and transfected using PEI reagent (Invitrogen) to form a virus packaging system. Viruses were collected twice after 48 and 72 h, centrifuged at 1000 rpm for 5 min, and purified with 0.45 μm syringe filter. Subsequently, stably transfected cells were established, SNU-739 and HCC-LM3 were incubated with lentivirus and medium supplemented with 10 μg/ml polybrene for 24 h in 6-well plates when 40%–50% fusion was reached. The transduced cells were screened with 2 μg/ml puromycin for 3 days, and when there were no surviving cells in the blank treatment group. The selected cells maintained a low concentration of puromycin during culture and identified by western blotting.

### Quantitative real-time PCR (qRT-PCR)

Total RNA was extracted from cells using Trizol Reagent (Invitrogen), followed by reverse transcription into cDNA using PrimeScript RT reagent (Takara). Detection of gene expression was performed using qRT-PCR method with SYBR Premix Ex Taq (Takara). The UBE2D1 primer sequences utilized were as follows: forward primer: 5′-TAGCGCATATCAAGGTGGAGT-3′, reverse primer: 5′-TGGTGACCATTGTG ACCTCAG-3′. β-actin and U6 were used for the normalization of UBE2D1 and miR-101, respectively. Fold change of expression was calculated as 2^−^^ΔΔCt^.

### Western blotting

Cells were lysed in RIPA lysis buffer (added protease and phosphatase inhibitors), and the protein concentration in the lysate was determined using a BCA assay kit. Protein lysates were resolved by SDS-PAGE with 10%–15% gel concentration based on the quality of the protein and transferred to nitrocellulose membranes. The membranes were blocked in 5% skim milk prepared in TBST buffer for 1 h and then incubated with primary antibodies against UBE2D1 (1:1000, Proteintech, 11677-1-AP), caspase 3 (1:1000, Cell Signaling Technology, #9662), γ-H2AX (1:2000, Abmart, M63324) and β-actin (1:2500, Cell Signaling Technology, #3700) overnight at 4 °C. Membranes were washed and incubated with HRP-conjugated secondary antibodies for 1 h at room temperature. The labeled bands were exposed to the ECL chemiluminescent reagent and imaged using a chemiluminescent imaging system.

### CCK8 assay

The proliferation of stable transfected SNU-739 and HCC-LM3 cell lines were detected via CCK8 assay. Each cell type was cultured in 96-well plates with 1 × 10^3^ cells/well in triplicate with continuous 0.5 μM drug treatment or no drug treatment, while setting up blank wells. Proliferative activity was measured every 24 h and maintained for 4 days. At the designated time, each well was added 10 μl CCK8 reagent and placed in 37 °C incubator for 1–4 h. The absorbance was then determined with a microplate reader at 450 nm. In the drug sensitivity test, SNU-739 and HCC-LM3 cells were treated with drugs at gradient concentrations, and the absorbance was detected after 48 h. Cell proliferation curves were created and analyzed using Excel and GraphPad Prism software.

### Colony formation assay

Each group was seeded with 1000 cells into 6-well plates by adjusting the density of the single-cell suspension. After the cells were grown overnight, the medium containing 0.5 μM drug was replaced and continued for 1–2 weeks. Until a single cell was cloned into a colony of more than 50 cells, the cell clones were washed with PBS and fixed with 4% paraformaldehyde, followed by staining with 0.5% (w/v) crystal violet dye (Sigma-Aldrich). Images of cell clones were captured by the Odyssey scanner (LI-COR, Lincoln, NE, USA) and then counted using the Image J software.

### Measurement of apoptosis

CDDP and 5Fu were added to the cell medium at a final concentration of 5 µM to treat the cells. After 24 h of incubation, cells were collected and stained with Annexin V/PI (Sigma-Aldrich) for 15 min at room temperature. Flow cytometry was used to carry out early and late cell apoptosis analysis.

### Immunofluorescence staining

Slides were sterilized prior to use, cells were grown on slides and treated with or without 10 μM cDDP or 5Fu for 2 h. Then the slides were washed and fixed with 4% paraformaldehyde. After permeabilized with 0.5% Triton X-100 for 15 min, slides were blocked for 1 h with 1% (w/v) BSA in PBS. This was followed by overnight incubation with primary γ-H2AX antibody (1:400, Abmart, M63324), and incubated with goat anti-mouse secondary antibody conjugated with FITC for 1 h. The nuclei were stained with DAPI (Sigma-Aldrich) for a duration of 5 min at room temperature. Expression levels of γ-H2AX were evaluated based on the number of positively stained foci per cell.

### Comet assay

Cells were treated with or without 5 μM cDDP or 5Fu for 12 h, and samples were collected through digestion and centrifugation. According to the instructions, 1% normal melting point agarose gel and 0.7% low melting point agarose gel were first prepared. Mixing 10,000 cells into a low melting point agarose gel and spread evenly on a slide, which is pre-coated with the first layer of normal melting point gel, and the third layer of low melting point agarose gel as protection. After cell lysis, the DNA was uncoiled under alkaline buffer, and the single-cell gel electrophoresis (~0.75–1 V/cm) should be performed in an ice bath and protected from light. Then the DNA was neutralized with neutral buffer, stained with PI, and DNA tailing was visualized under red fluorescence by fluorescence microscopy.

### Dual-luciferase reporter assay

A fragment of the UBE2D1 3′UTR containing the putative binding site of miR-101 was subcloned into pGL3-Enhancer Vector containing the firefly luciferase gene to construct the UBE2D1 wild-type (WT) or mutant (MUT) reporter plasmid. The phRL-TK vector containing Renilla luciferase gene was used as an internal control. HEK293 cells were seeded in 48-well plates at 70% cell confluence, followed by co-transfected with the above luciferase plasmids (5 ng of plasmids encoding Renilla luciferase and 100 ng of plasmids containing firefly luciferase reporters) and 20 ng miR-101 mimics using Lipofectamine 2000. 48 h after transfection, the cells were lysed and luciferase activity was detected using the Dual-Luciferase Reporter assay kit (Promega). Reporter luciferase activity was normalized to its respective Renilla luciferase activity for each transfected well. All values were expressed as fold change over control.

### MiRNA and transfection

MiR-101 mimics were chemically synthesized and purified by GenePharma (Shanghai, China), which mimic naturally occurring miR-101 after transfection of cells. The synthesized miR-101 inhibitors specifically inhibited the function of miR-101 when transfected into cells. The sense strand sequences of miR-101 mimics, miR-101 inhibitors and their negative controls are listed in Table S3. According to the manufacturer’s instructions, 20 μM miR-101 mimics or miR-101 inhibitors were transfected into cells with liposome reagent 2000 in a 1:1 ratio.

### Treatment of xenograft with cDDP

The animal experiments conducted in this study were approved by the Committee for Experimental Animal Use and Care of the Fourth Military Medical University. All experimental procedures adhered to the National Guidelines for Animal Experimentation. The 5-week-old male nude mice used in this experiment were obtained from the Experimental Animal Center of the Fourth Military Medical University. The xenograft models were generated by injecting SNU-739-shUBE2D1 or control cells subcutaneously in the flank of nude mice. A suspension of 5 × 10^6^ tumor cells in 100 μl PBS was mixed with 100 μl matrigel. When the tumor volume reached 150 mm^3^ and grow normally, mice were randomly divided into four groups of three mice. For drug intervention, mice were administered an intraperitoneal injection of 2 μM of the chemotherapeutic drug cDDP for every 3 days. Mouse body weight and tumor size were recorded every 3 days during the period. The tumor volume was calculated according to the formula: *V* = (length × width^2^)/2. Following which animals were euthanized, the tumors were harvested and weighed, then fixed with 4% paraformaldehyde for histological analyses.

### Immunohistochemistry

The fresh tumor samples were fixed in neutral buffered formalin for 24 h at room temperature and then embedded and processed according to standard protocols. The sections underwent deparaffinized through graded ethanol solutions and rehydration. After an antigen retrieval procedure of 30 min using antigen retrieval solution, the sections were stained with Ki67, γ-H2AX and TUNEL. The stained slices are scanned by the Servicebio company.

### Statistics

Experiments were repeated at least three times. Statistical analyses were performed using GraphPad Prism 8 software. The data in statistical graphs were presented as mean ± standard deviation (SD). Unless otherwise specified, group comparisons were assessed using unpaired Student’s *t*-test or one-way ANOVA. Survival curves were analyzed using the Log-rank test. The statistical significance of differences was determined at a threshold of *P* < 0.05.

## Supplementary information

Full and uncropped western blots

Supplemental Material

## Data Availability

The data that support the findings of this study are available on request from the corresponding author upon reasonable request.
