# Supplementary material for: Aberrant activation of a miR-101–UBE2D1 axis contributes to the advanced progression and chemotherapy sensitivity in human hepatocellular carcinoma
Source: Cell Death Discov. 2024 Oct 1;10:422. doi: 10.1038/s41420-024-02193-y (PMC11445525; doi:10.1038/s41420-024-02193-y)
Supplement: Supplementary file 1 — Full and uncropped western blots [file 41420_2024_2193_MOESM1_ESM.docx]

Fig 3A

SNU-739

Grouped into: control、shUBE2D1-1、shUBE2D1-2

Left band: UBE2D1 (17kD)

Right band: β-actin (45kD)


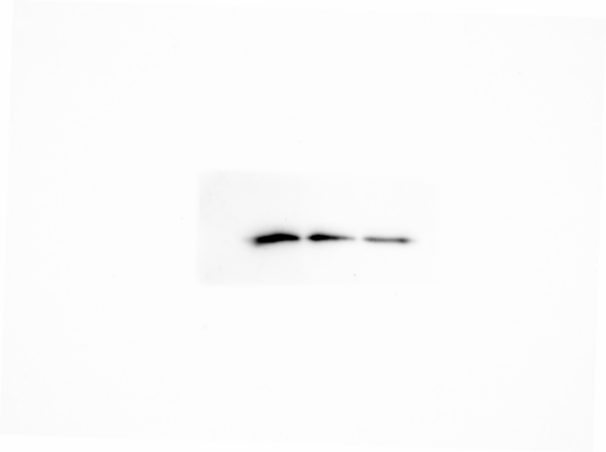

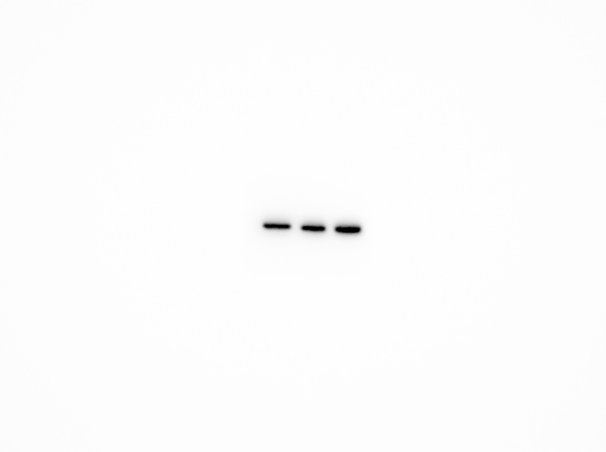


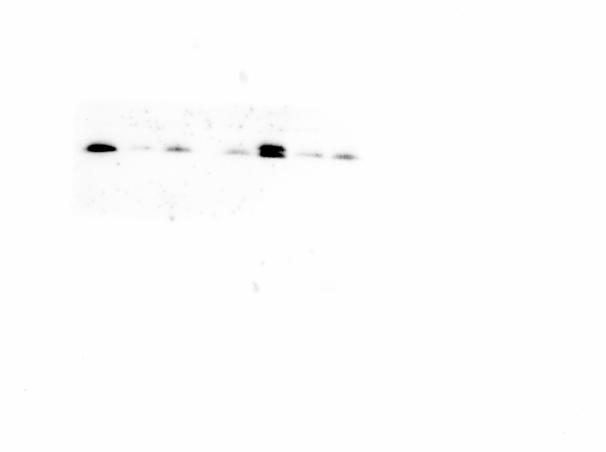

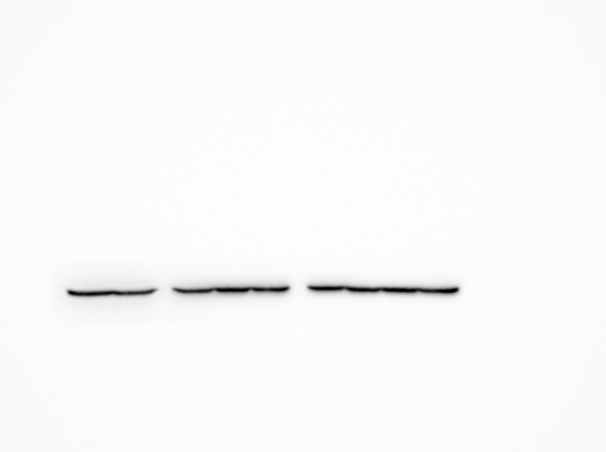

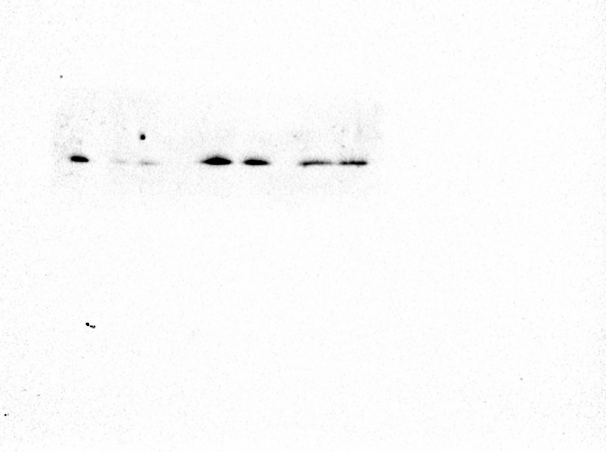

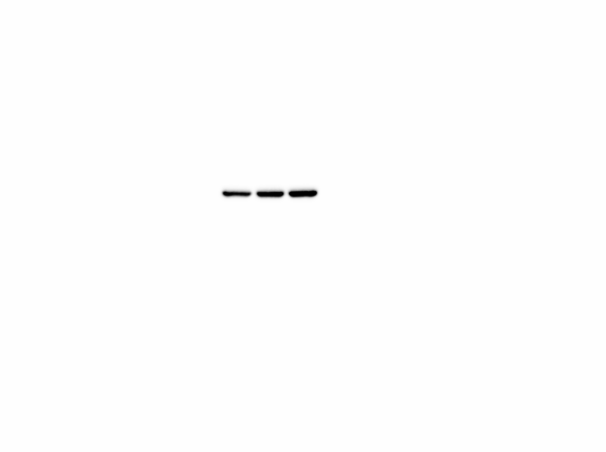


Fig 3A

HCC-LM3

Grouped into: control、shUBE2D1-1、shUBE2D1-2

Left band: UBE2D1 (17kD)

Right band: β-actin (45kD)


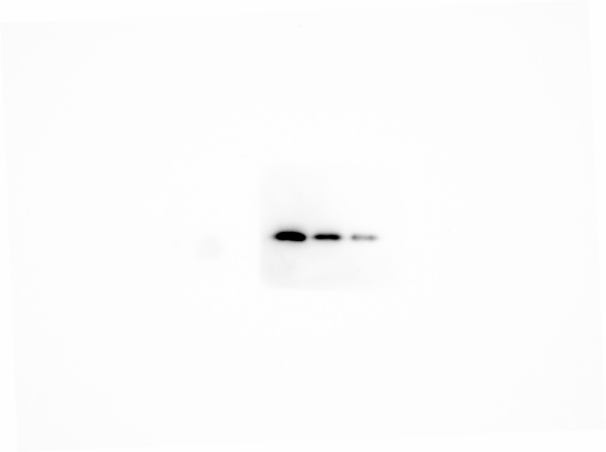

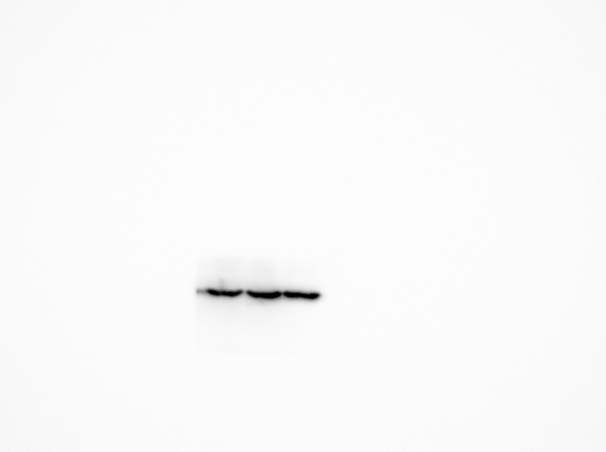


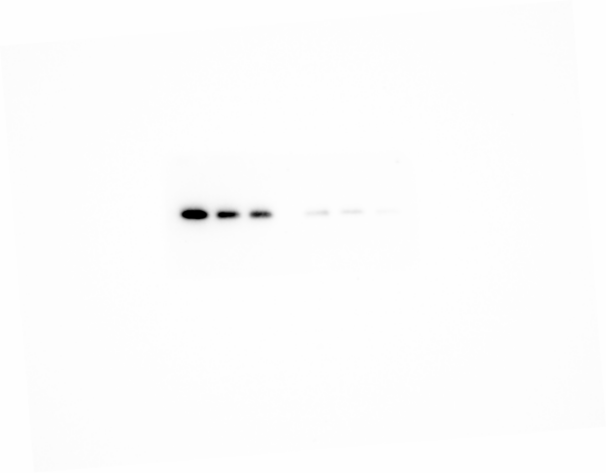

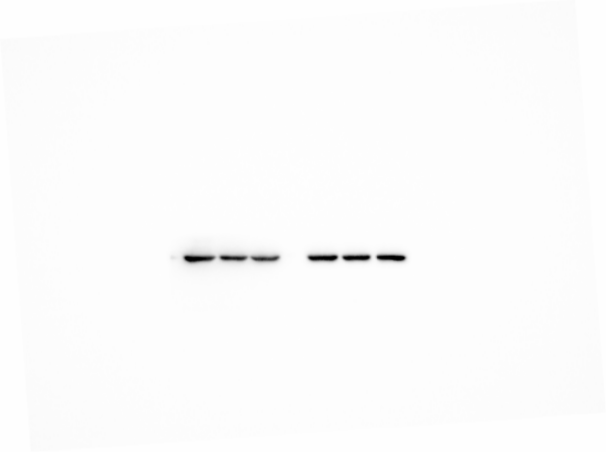


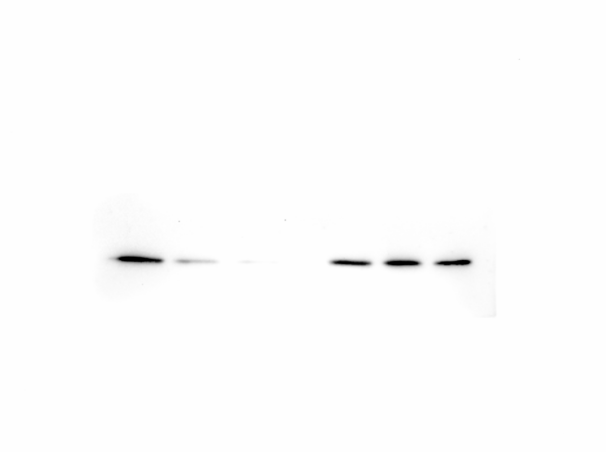

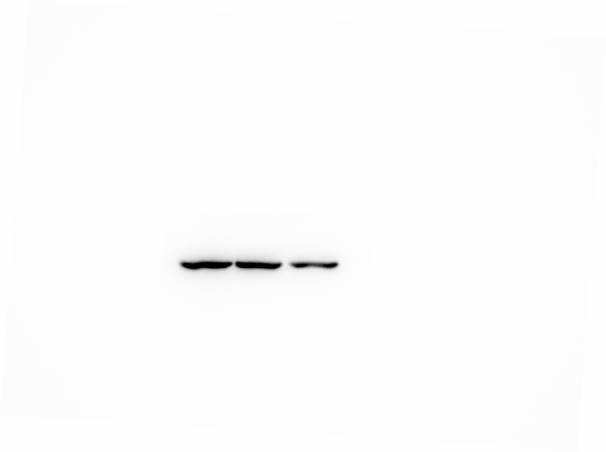


Fig 3F

SNU-739

Grouped into: control、shUBE2D1-1、shUBE2D1-2、control+5Fu、shUBE2D1-1+5Fu、shUBE2D1-2+5Fu

Left band: cleaved caspase3 (17kD)

Right band: β-actin (45kD)


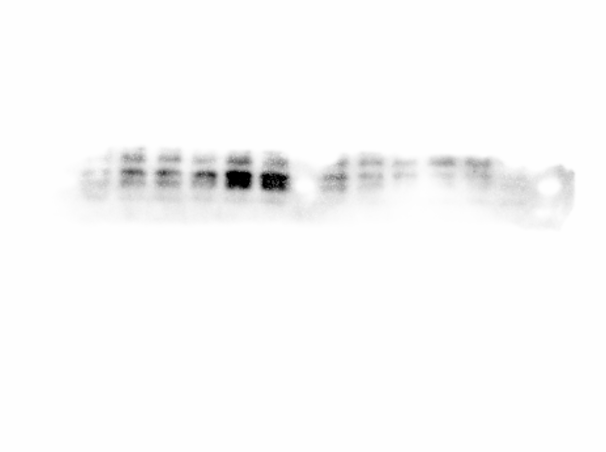

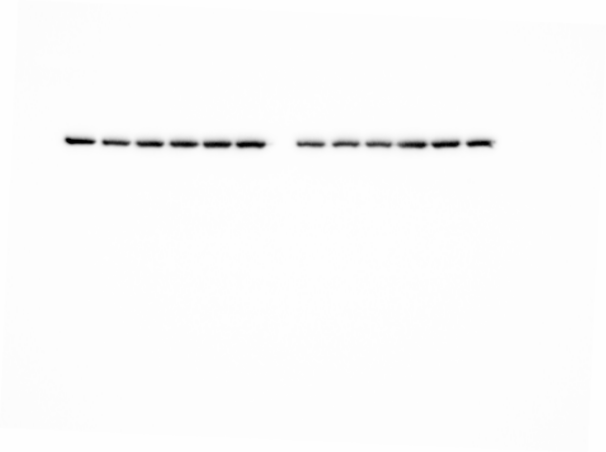


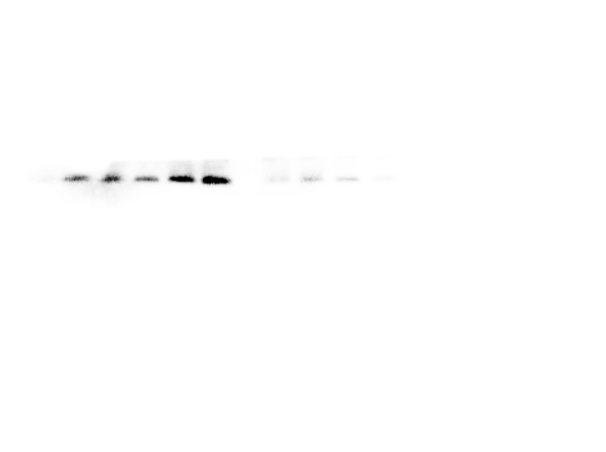

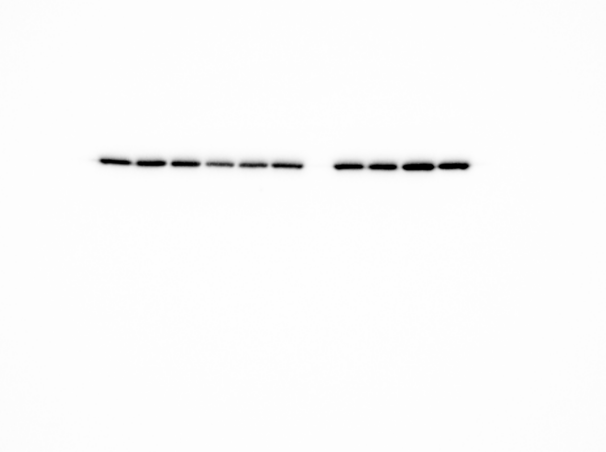


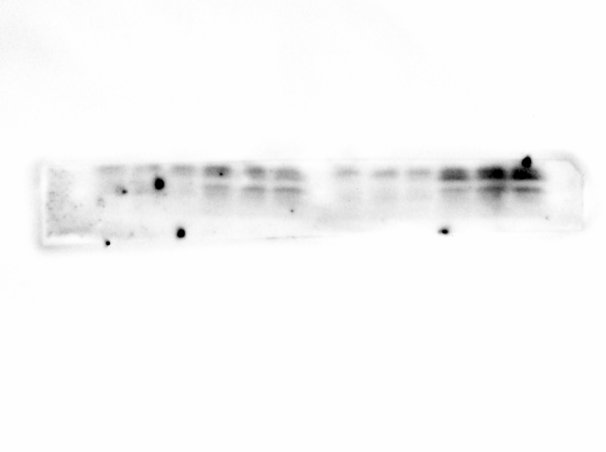

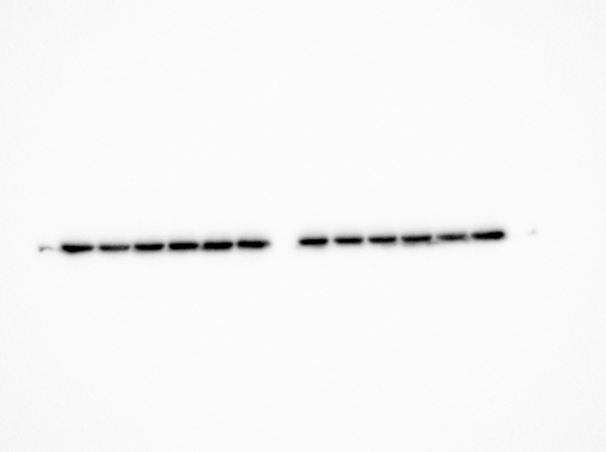


Fig 3F

HCC-LM3

Grouped into: control、shUBE2D1-1、shUBE2D1-2、control+cDDP、shUBE2D1-1+cDDP、shUBE2D1-2+cDDP

Left band: cleaved caspase3 (17kD)

Right band: β-actin (45kD)


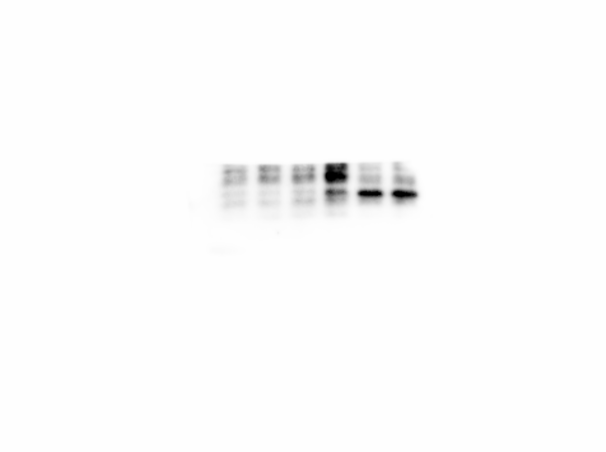

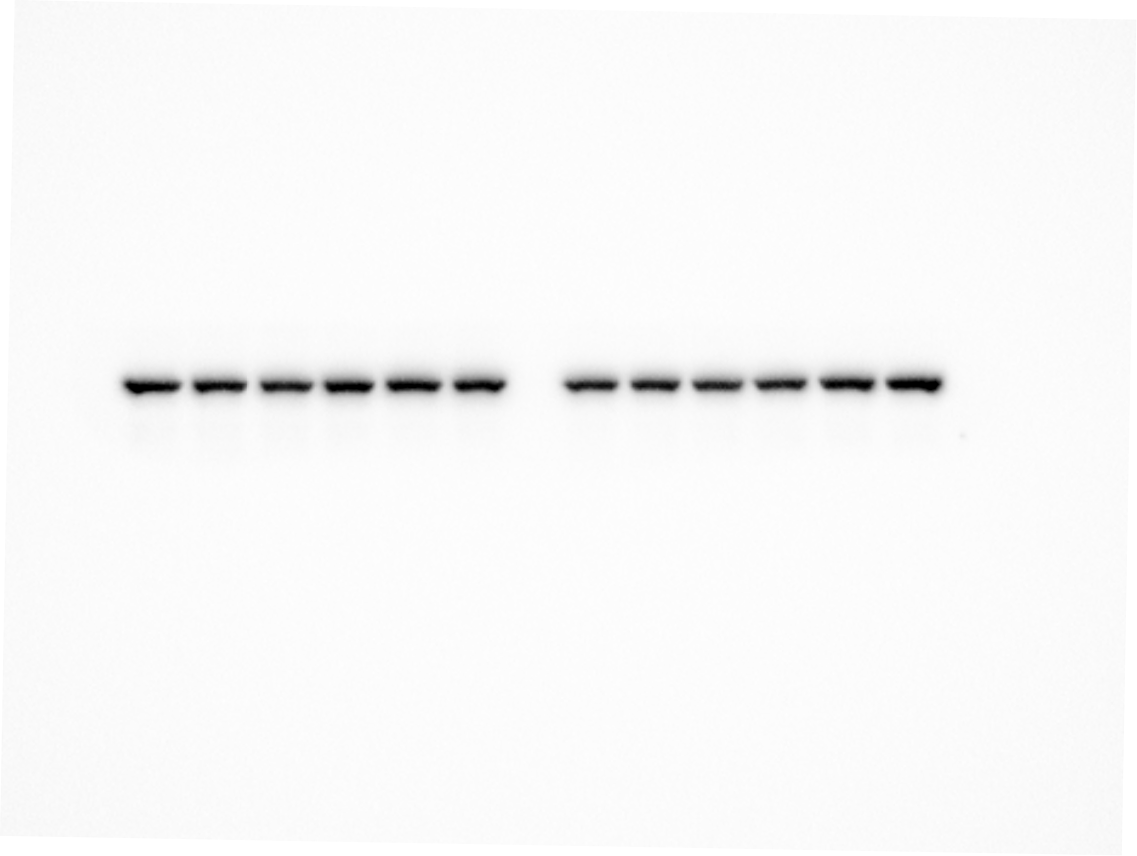


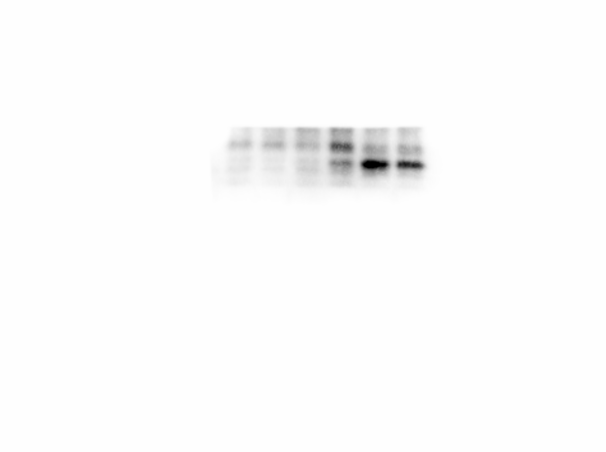

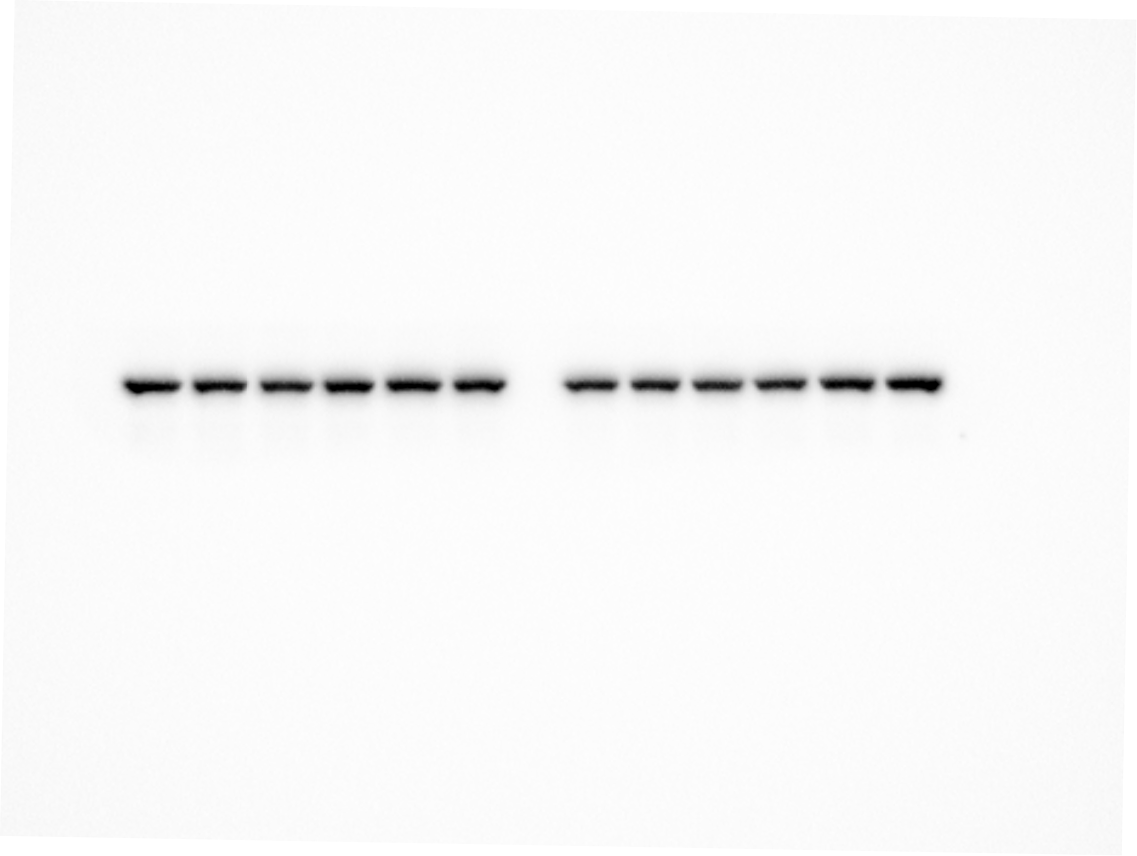


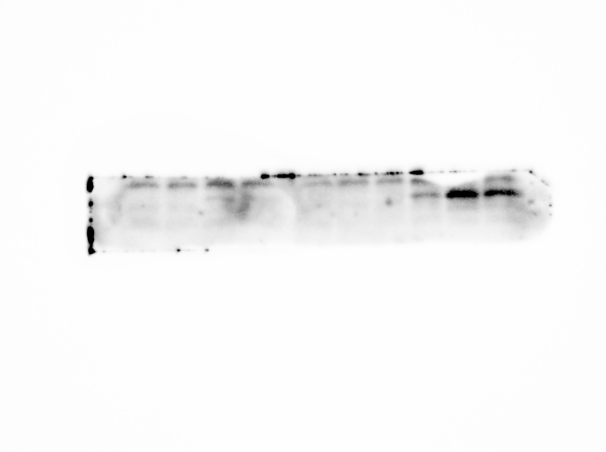

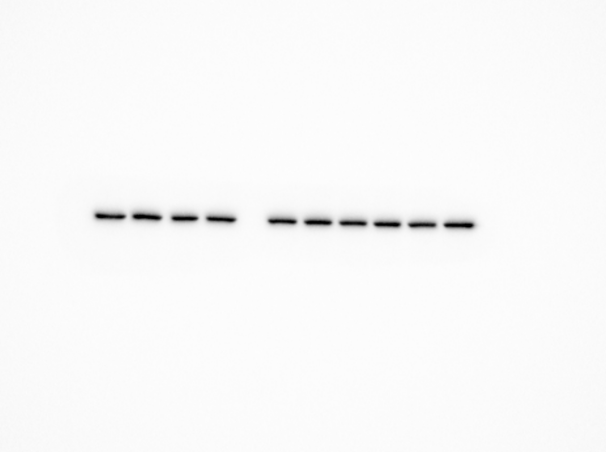


Fig 4A

SNU-739

Grouped into: control、OE-UBE2D1

Left band: UBE2D1 (17kD)

Right band: β-actin (45kD)


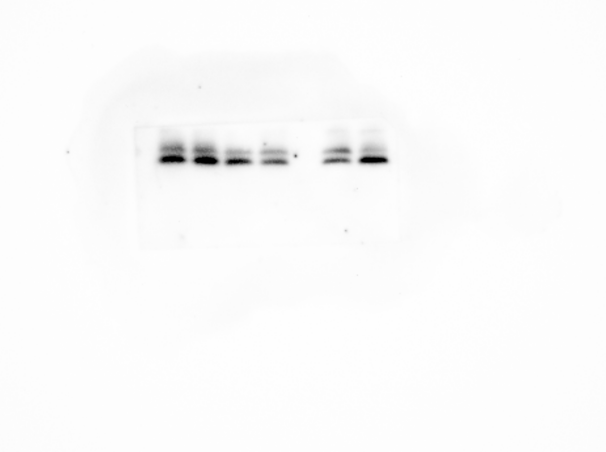

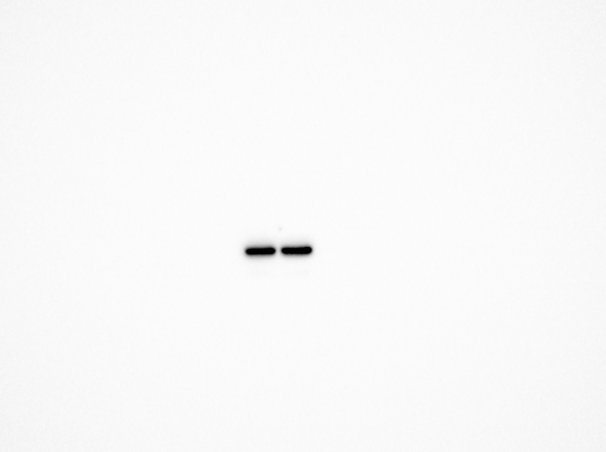


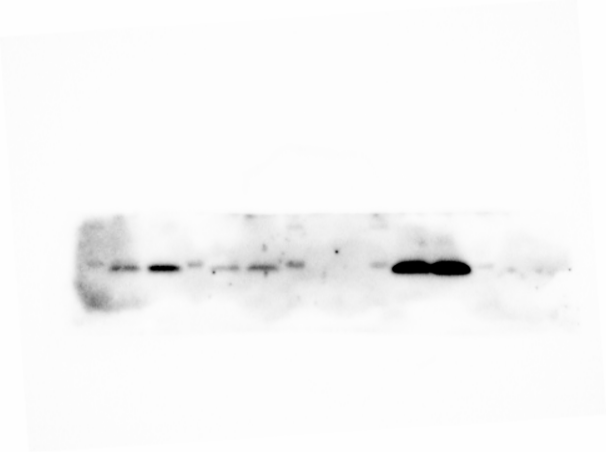

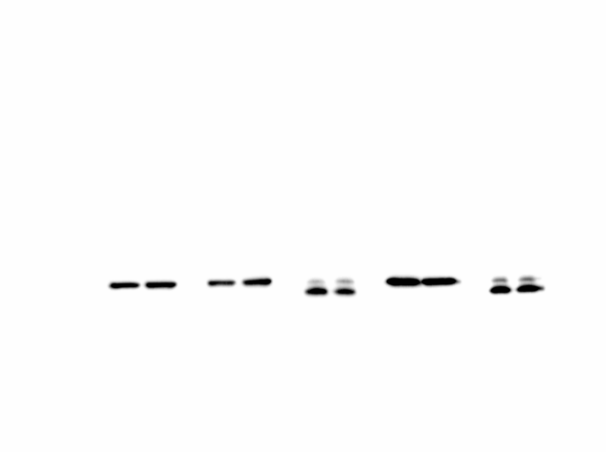


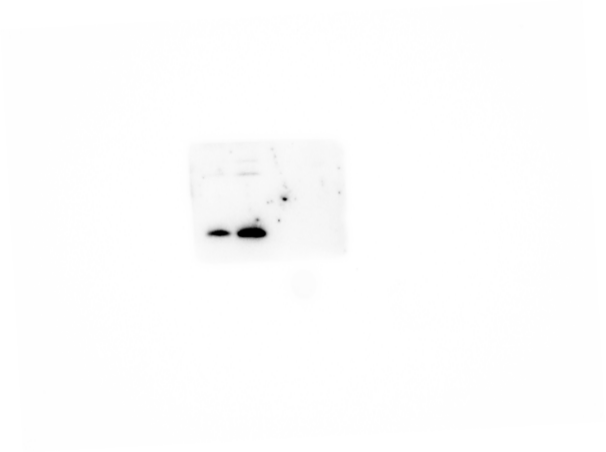

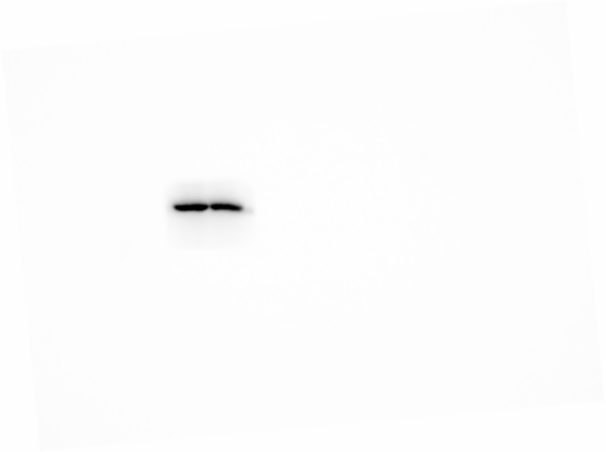


Fig 4A

HCC-LM3

Grouped into: control、OE-UBE2D1

Left band: UBE2D1 (17kD)

Right band: β-actin (45kD)


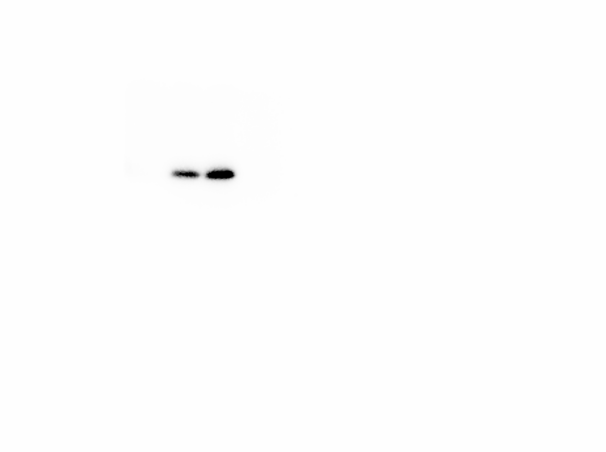

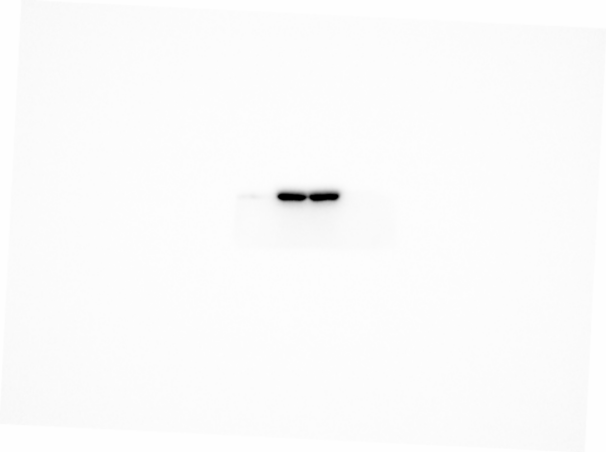


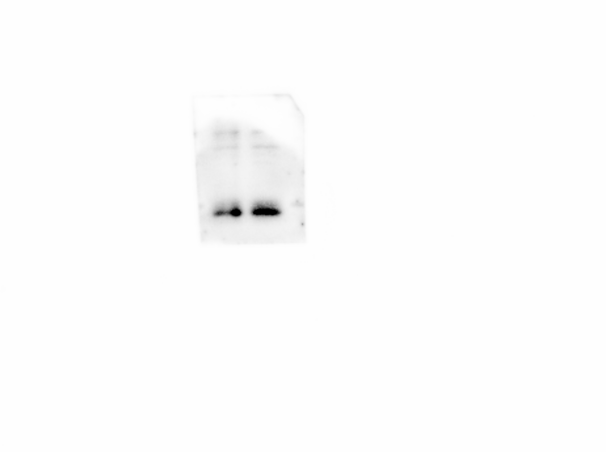

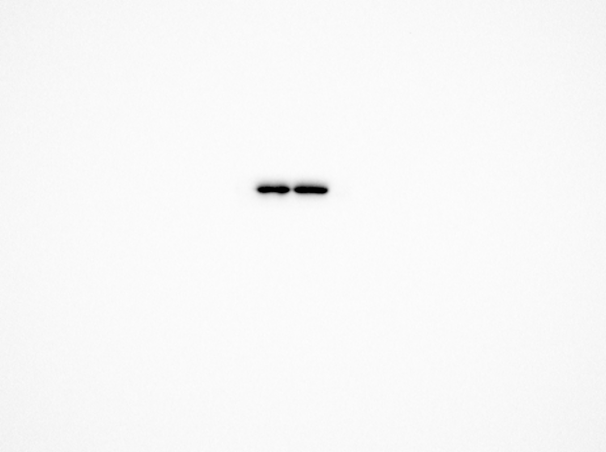


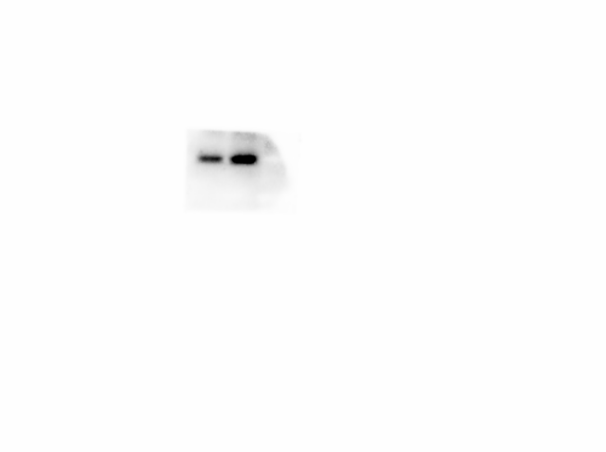

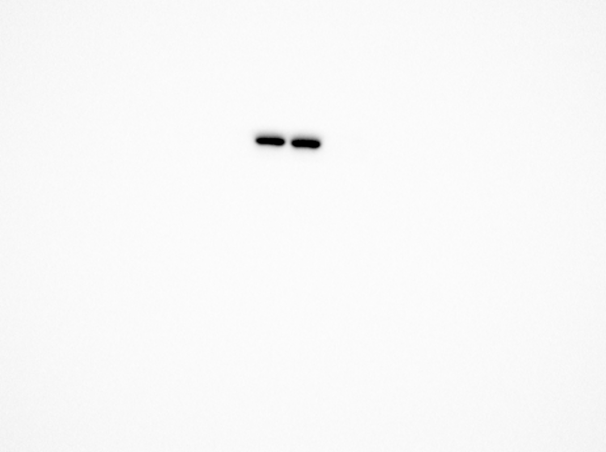


Fig 4F

SNU-739

Grouped into: control、OE-UBE2D1、control+cDDP、OE-UBE2D1+cDDP

Left band: cleaved caspase3 (17kD)

Right band: β-actin (45kD)


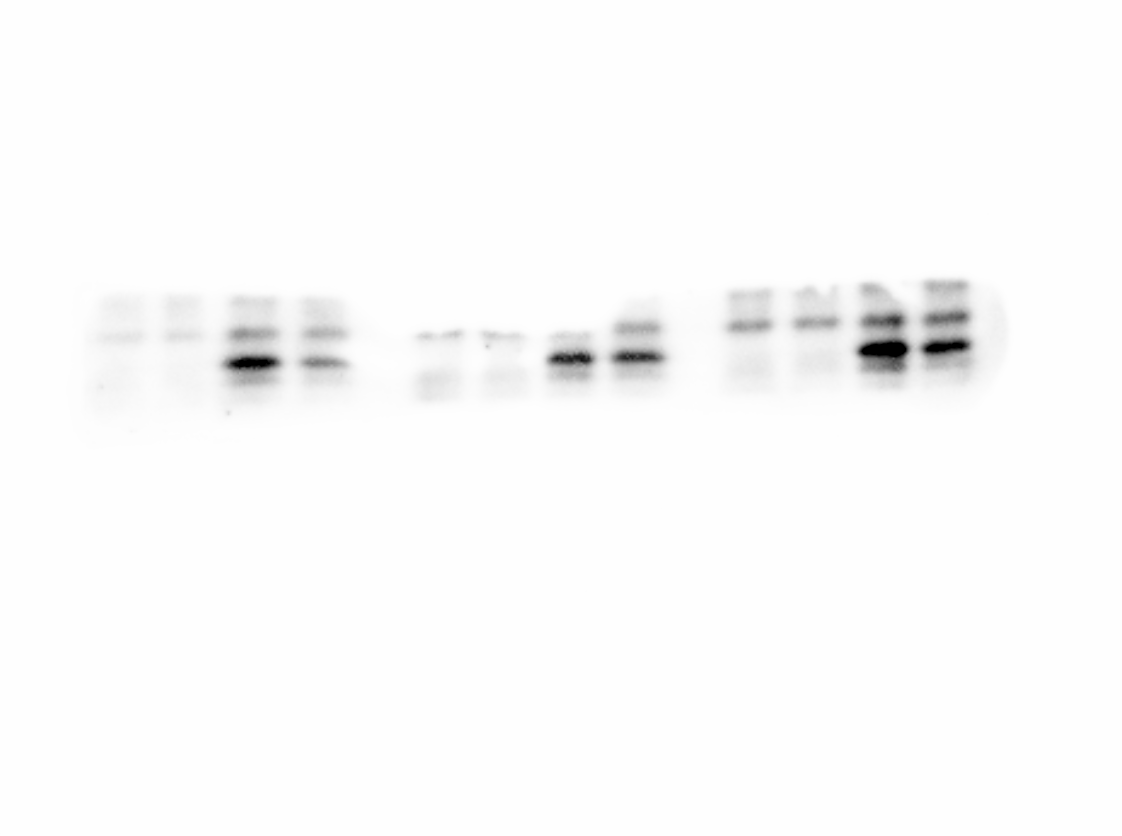

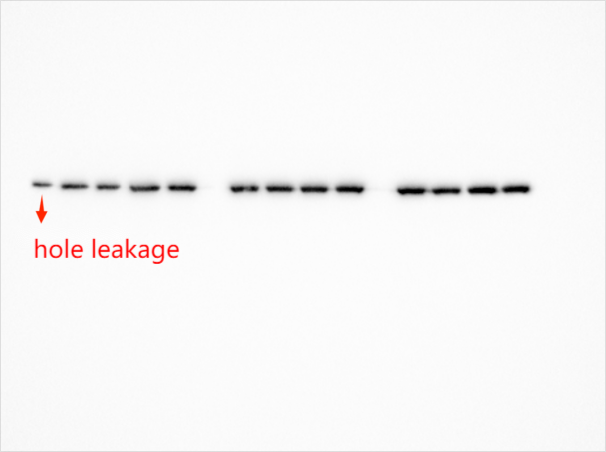


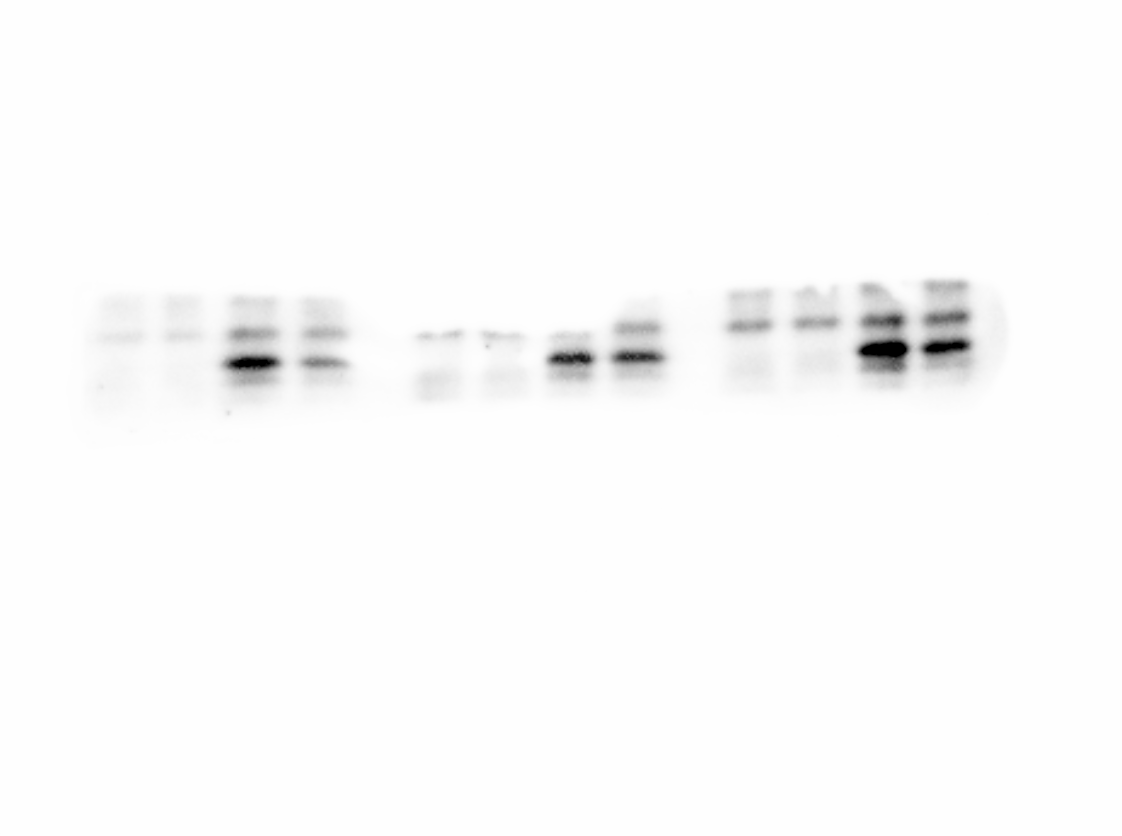

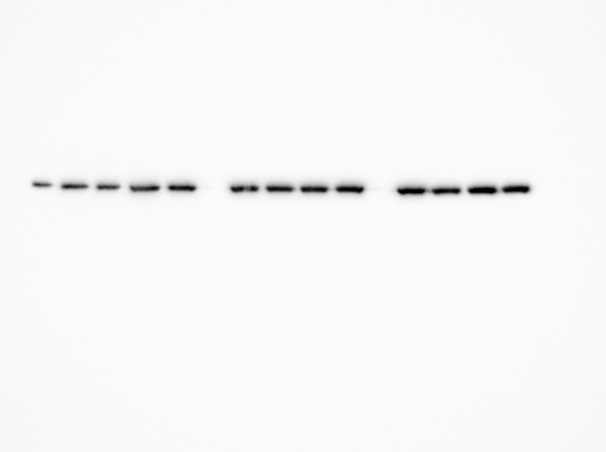


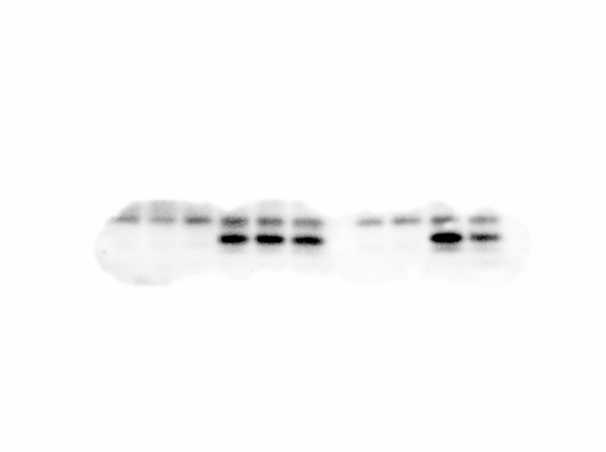

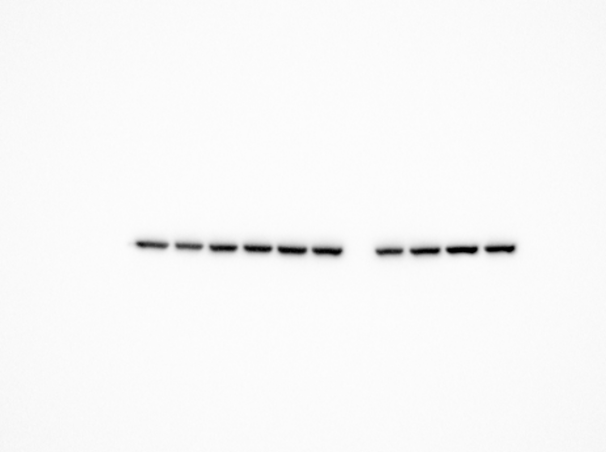


Fig 4F

HCC-LM3

Grouped into: control、OE-UBE2D1、control+5Fu、OE-UBE2D1+5Fu

Left band: cleaved caspase3 (17kD)

Right band: β-actin (45kD)


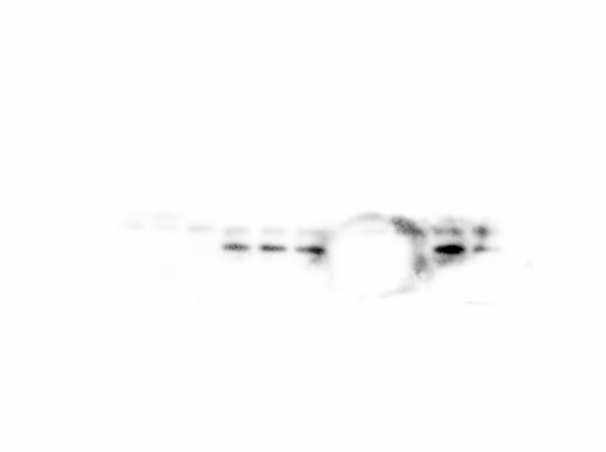

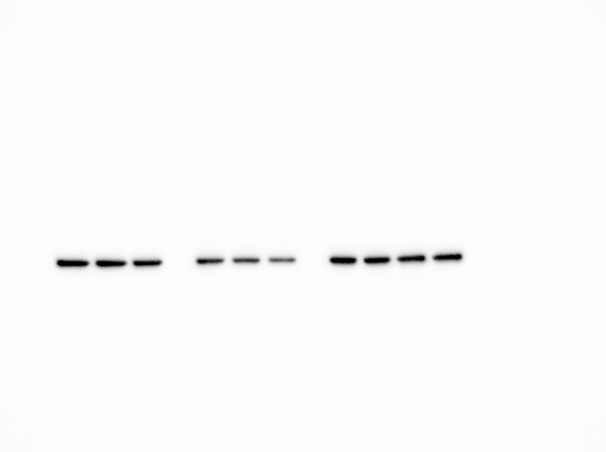


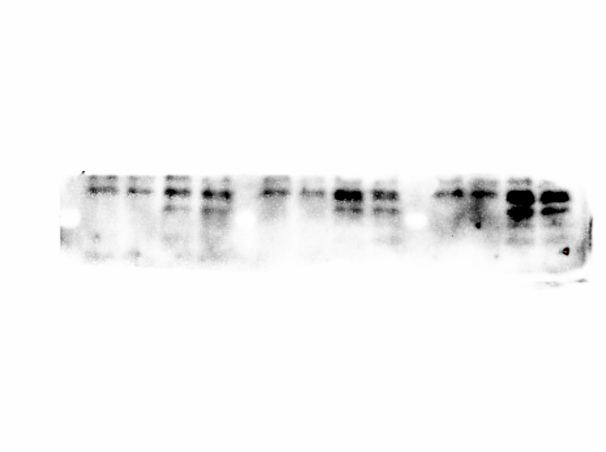

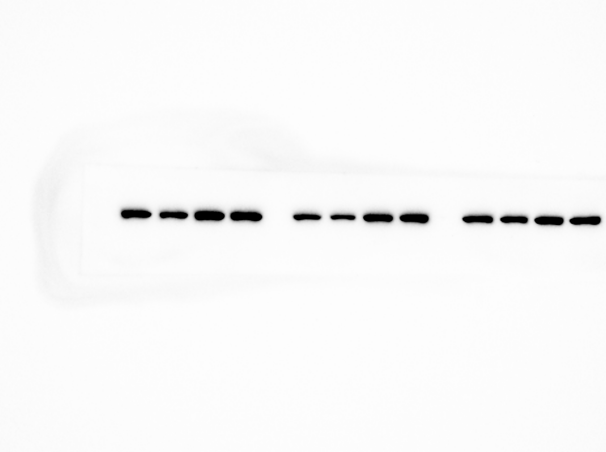


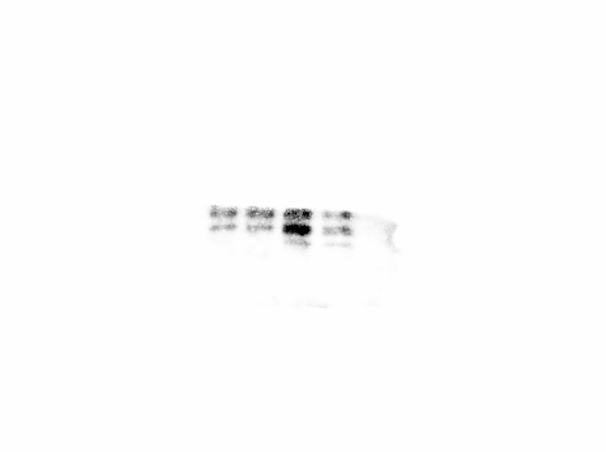

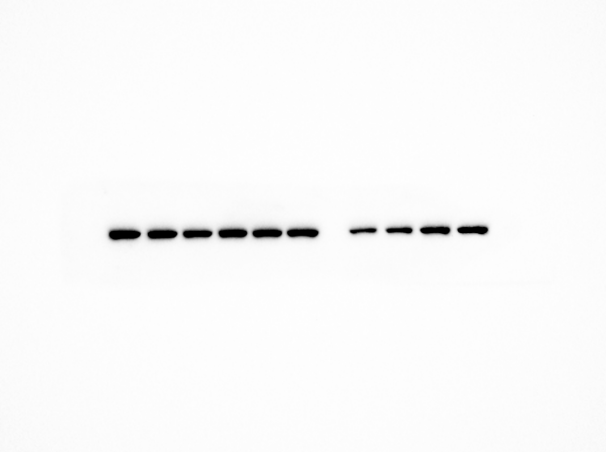


Fig 5A

SNU-739

Grouped into: control、shUBE2D1-1、shUBE2D1-2、control+5Fu、shUBE2D1-1+5Fu、shUBE2D1-2+5Fu

Left band: γ-H2AX (15kD)

Right band: β-actin (45kD)


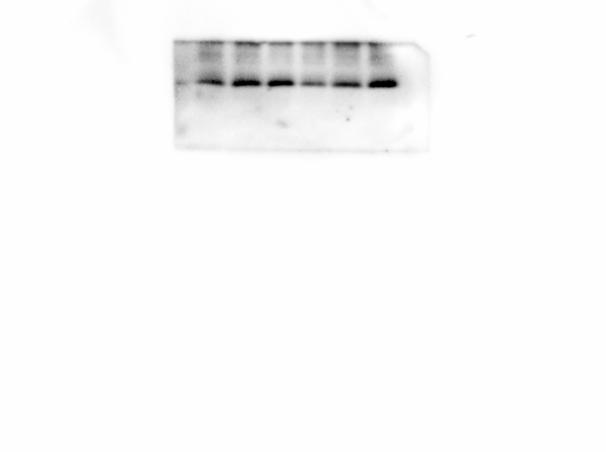

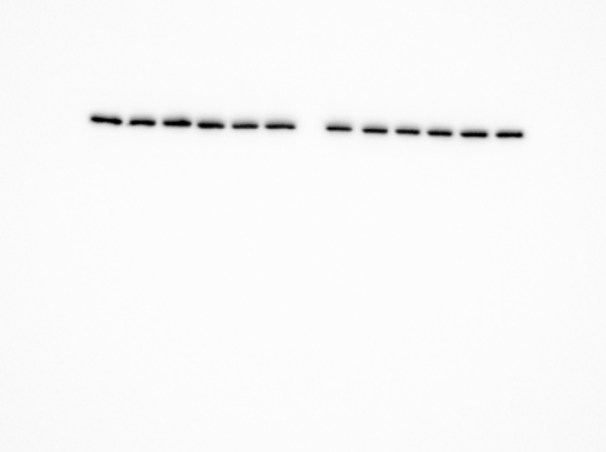


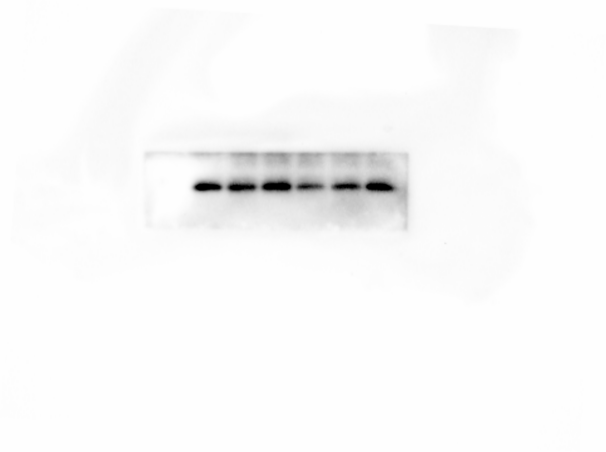

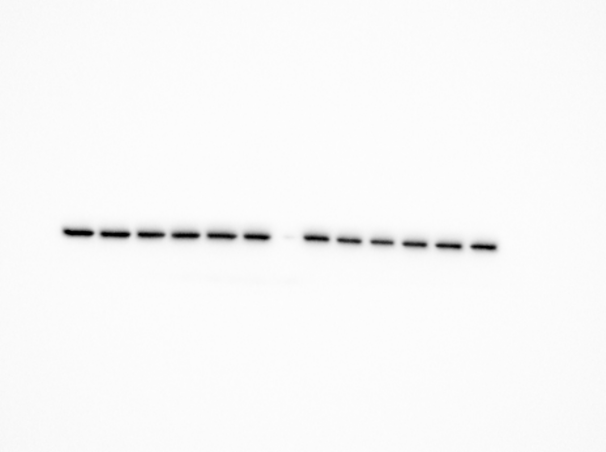


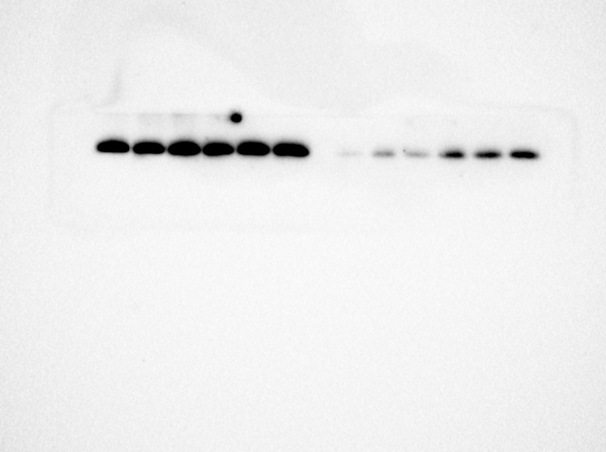

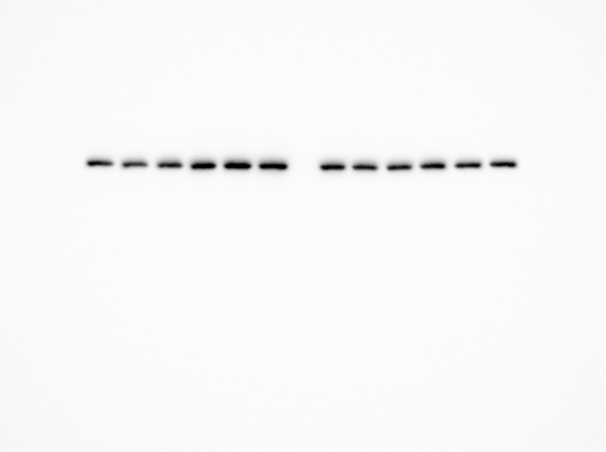


Fig 5A

HCC-LM3

Grouped into: control、shUBE2D1-1、shUBE2D1-2、control+cDDP、shUBE2D1-1+cDDP、shUBE2D1-2+cDDP

Left band: γ-H2AX (15kD)

Right band: β-actin (45kD)


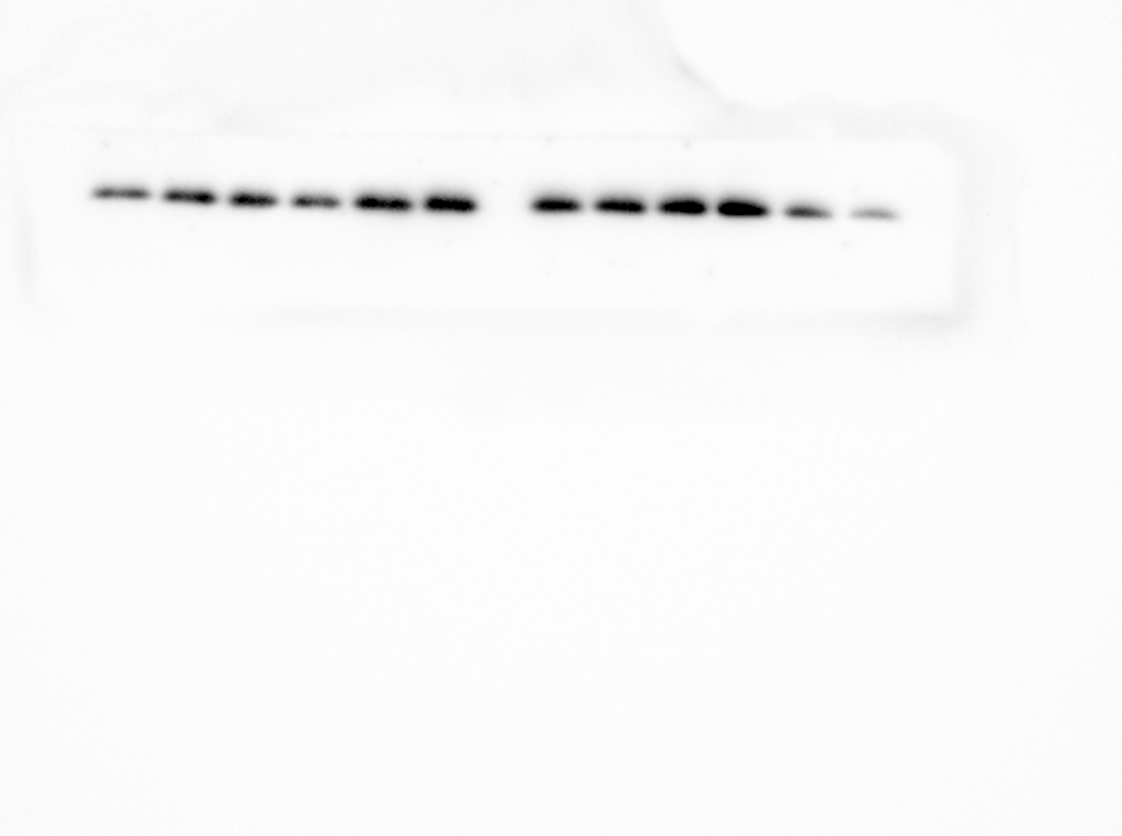

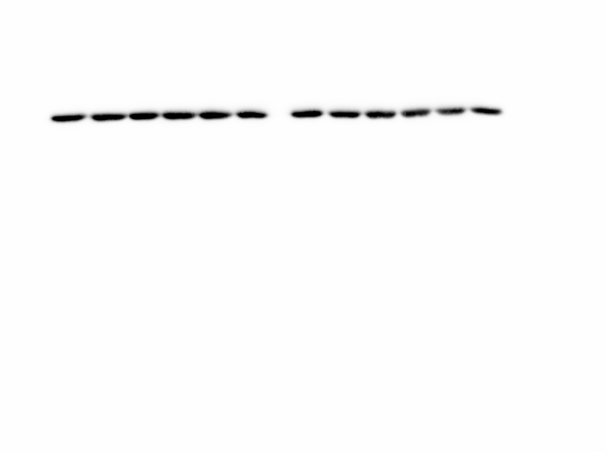


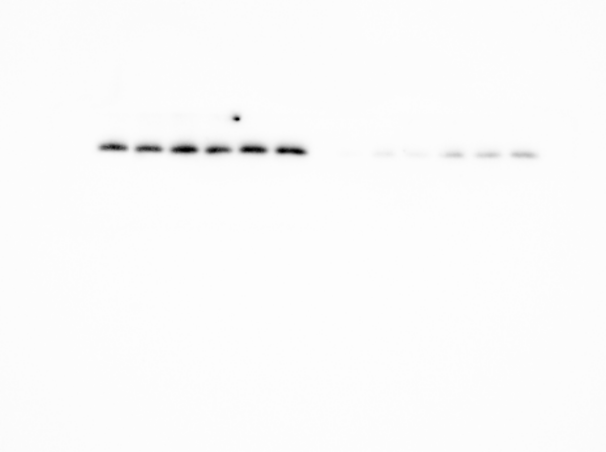

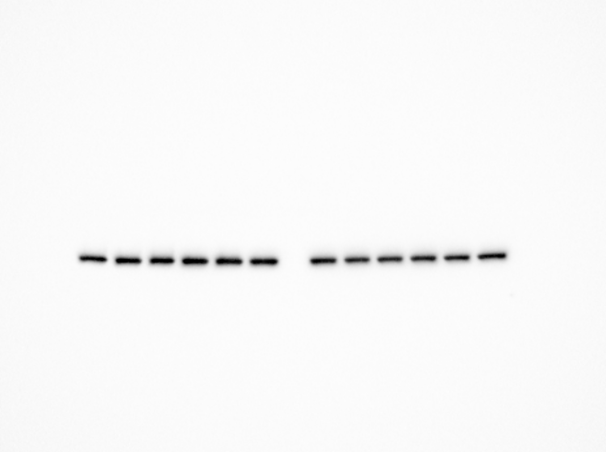


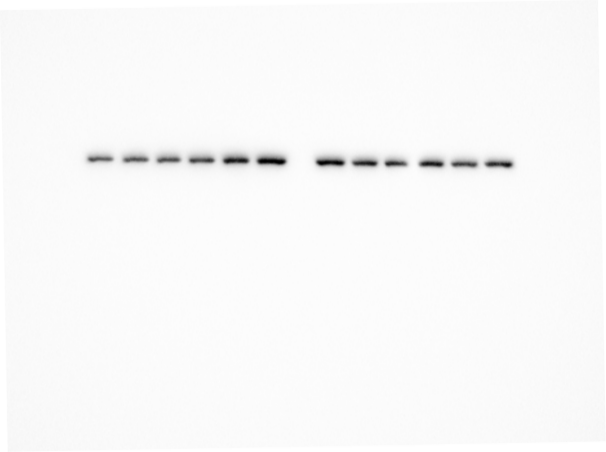

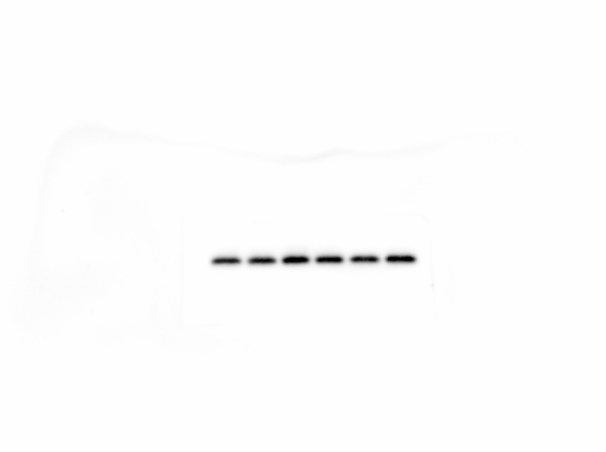


Fig 5D

SNU-739

Grouped into: control、OE-UBE2D1、control+cDDP、OE-UBE2D1+cDDP

Left band: γ-H2AX (15kD)

Right band: β-actin (45kD)


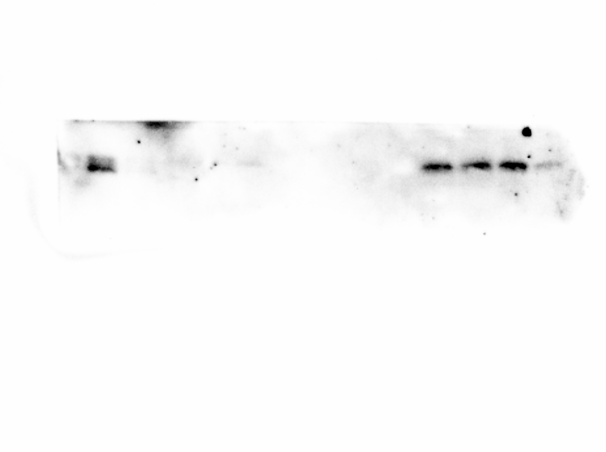

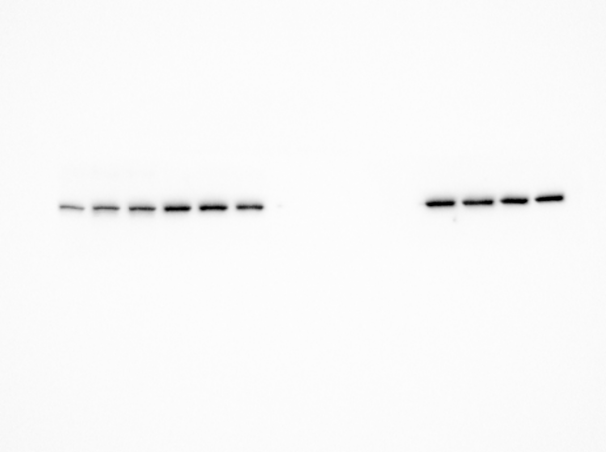


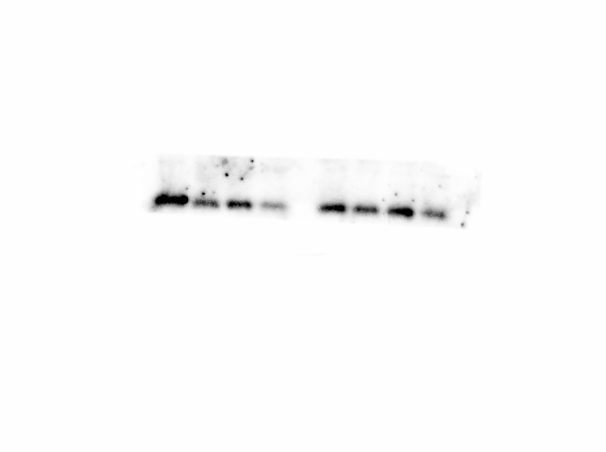

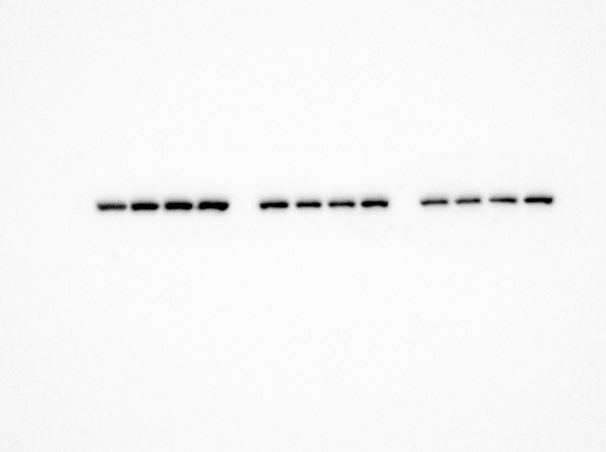


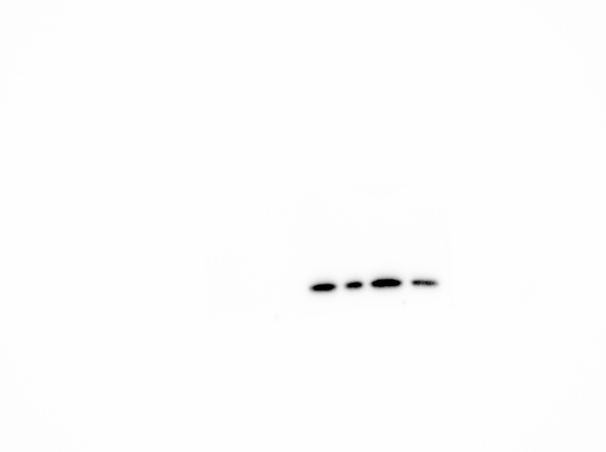

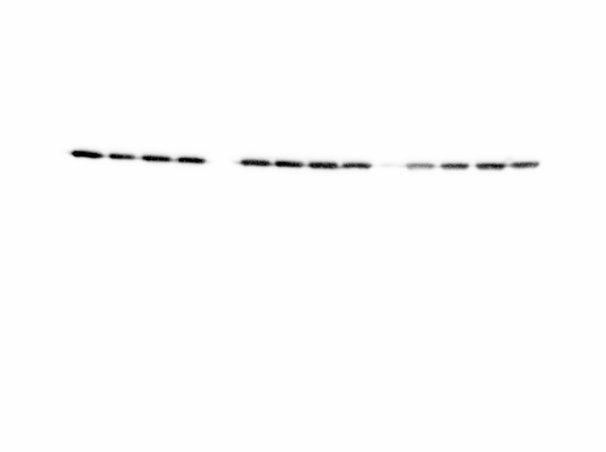


Fig 5D

HCC-LM3

Grouped into: control、OE-UBE2D1、control+5Fu、OE-UBE2D1+5Fu

Left band: γ-H2AX (15kD)

Right band: β-actin (45kD)


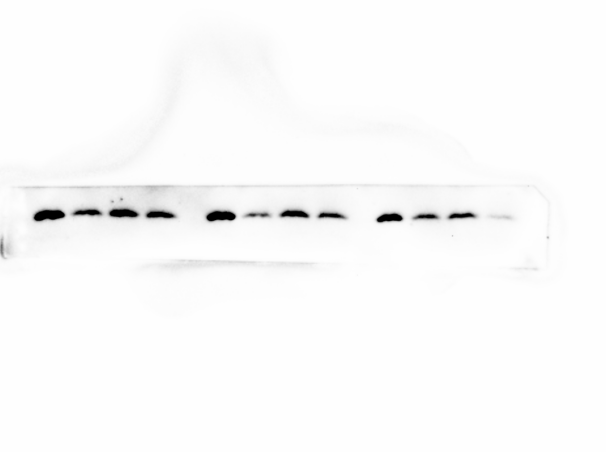

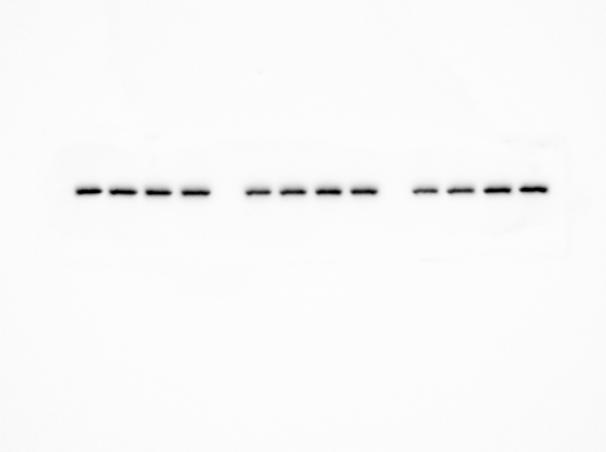


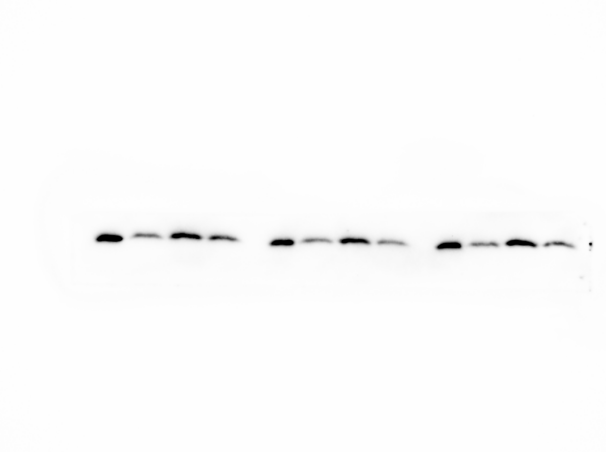

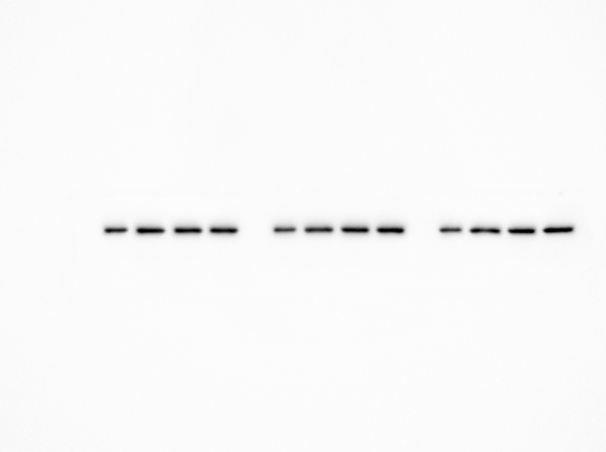


Fig 6C

SNU-739

Grouped into: NC mimics、miR101 mimics

Left band: UBE2D1 (17kD)

Right band: β-actin (45kD)


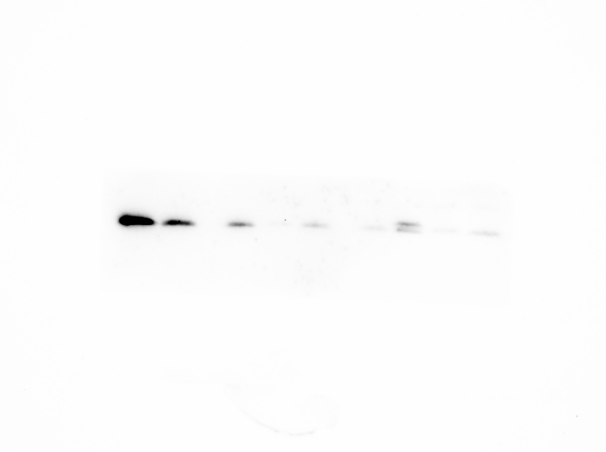

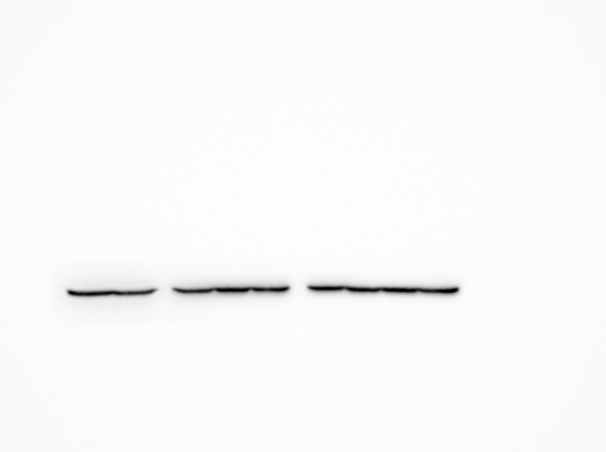


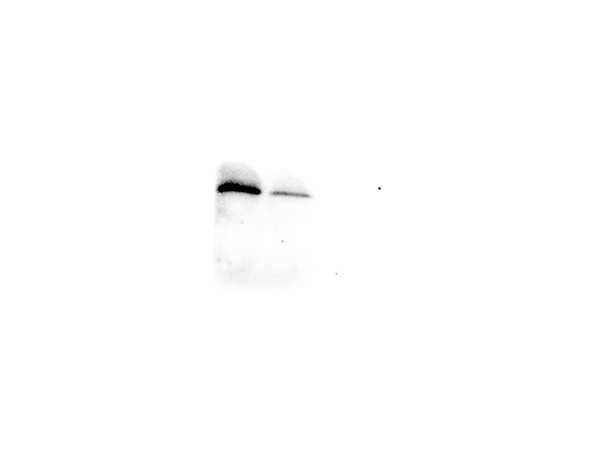

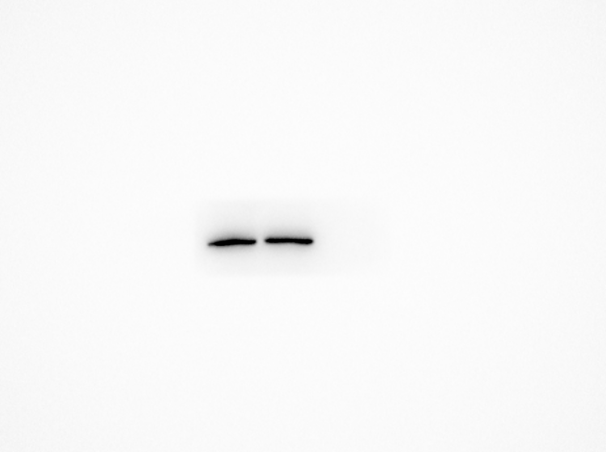


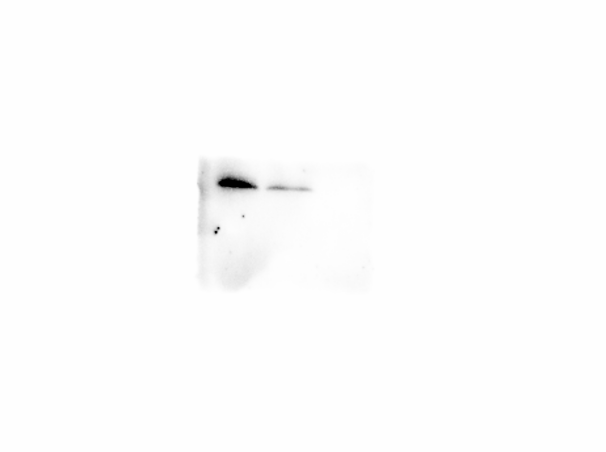

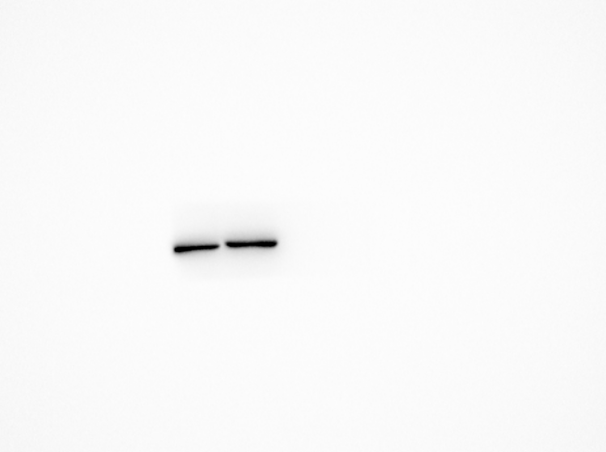


Fig 6C

HCC-LM3

Grouped into: NC mimics、miR101 mimics

Left band: UBE2D1 (17kD)

Right band: β-actin (45kD)


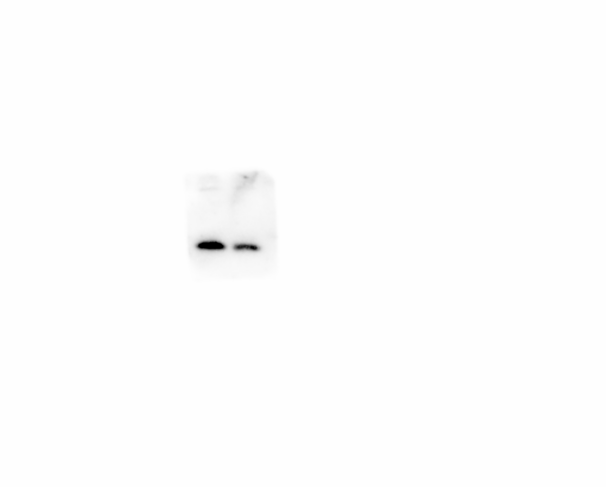

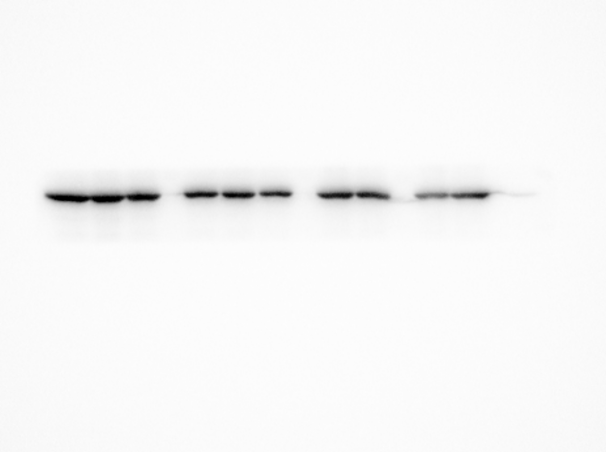


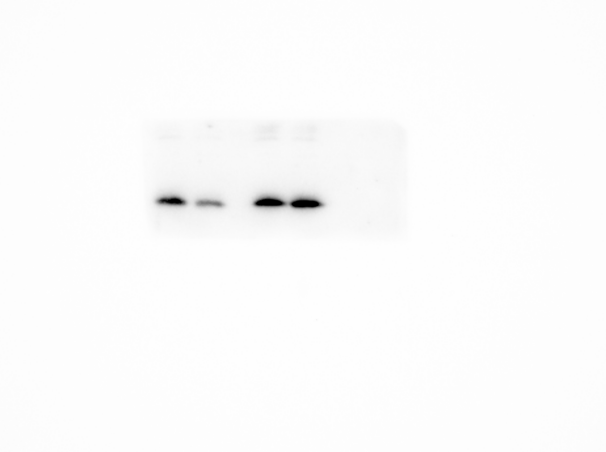

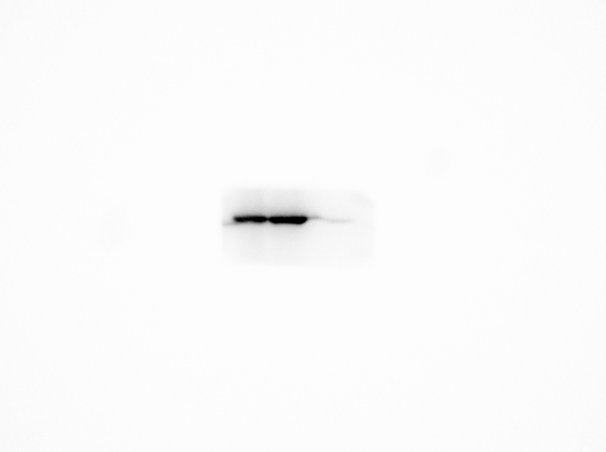


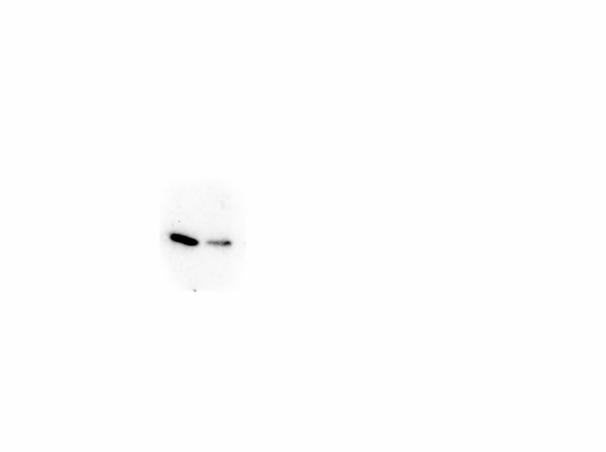

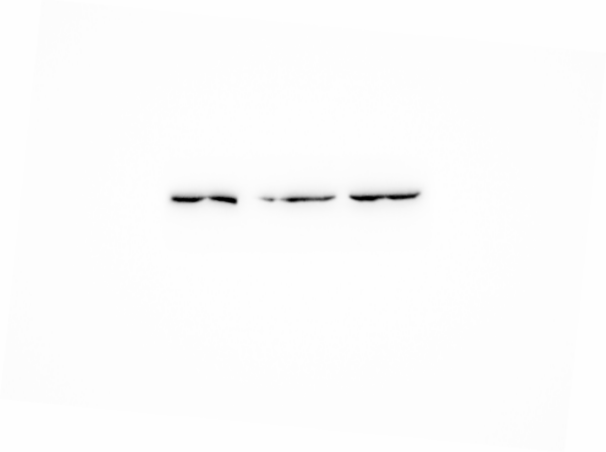


Fig 6D

SNU-739

Grouped into: NC inhibitors、miR101 inhibitors

Left band: UBE2D1 (17kD)

Right band: β-actin (45kD)


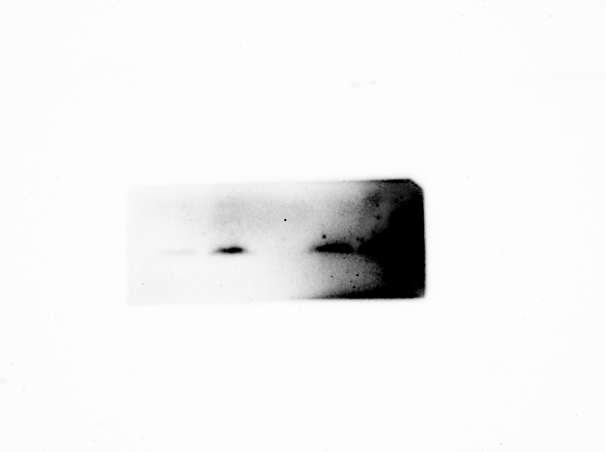

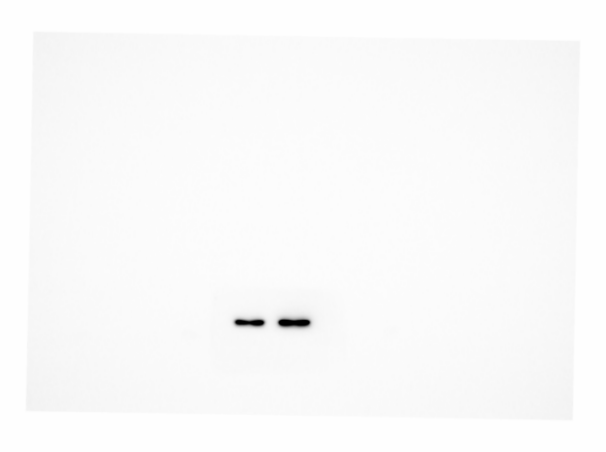


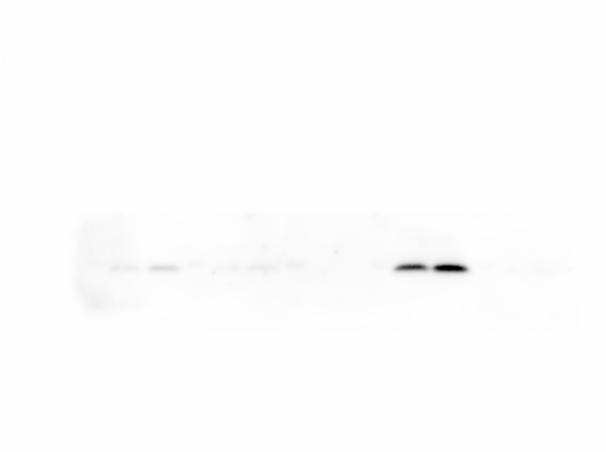

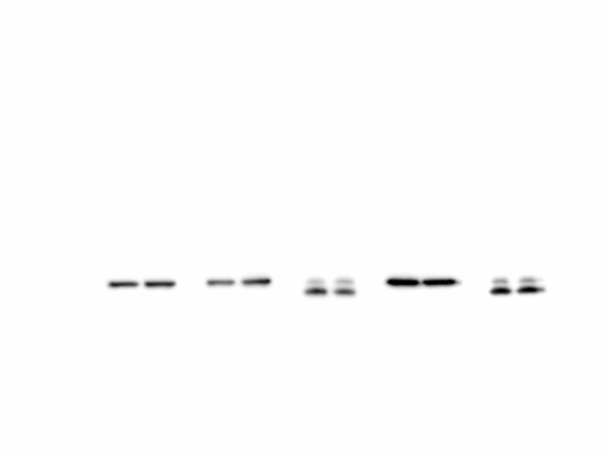


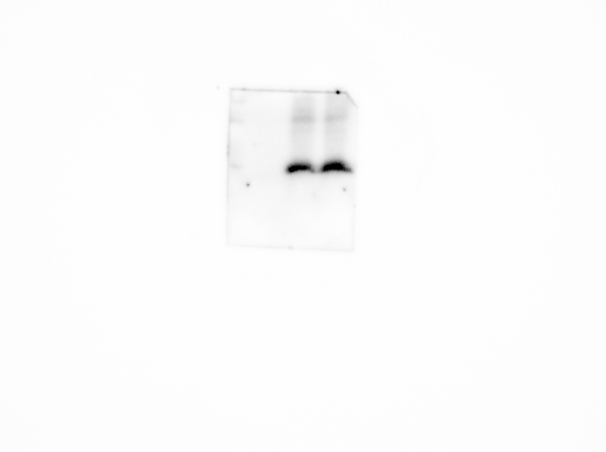

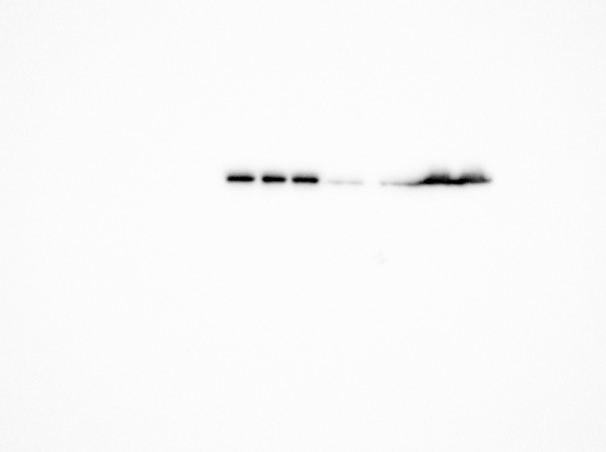


Fig 6D

HCC-LM3

Grouped into: NC inhibitors、miR101 inhibitors

Left band: UBE2D1 (17kD)

Right band: β-actin (45kD)


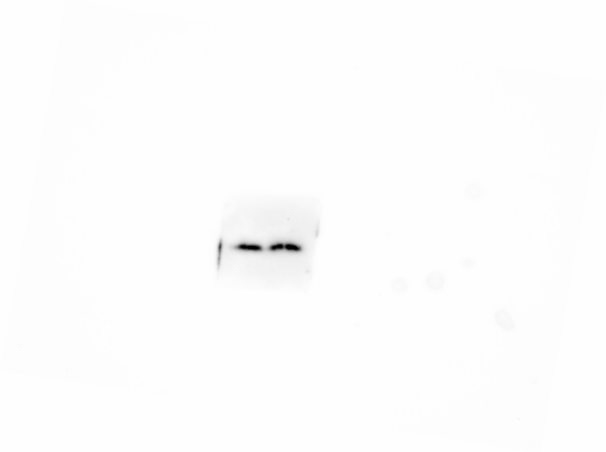

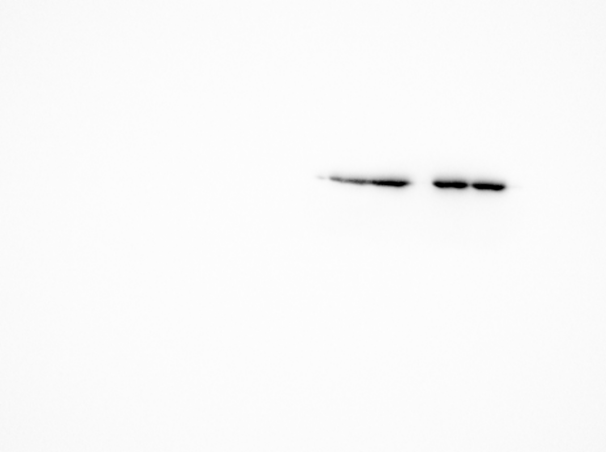


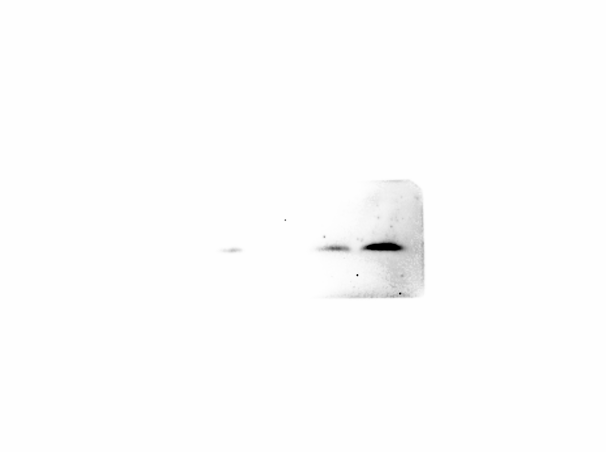

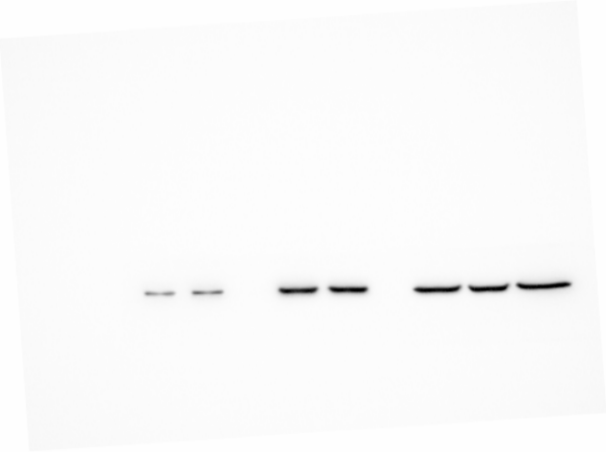


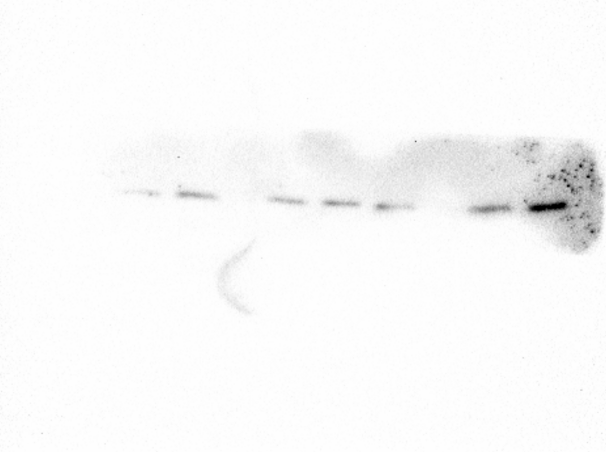

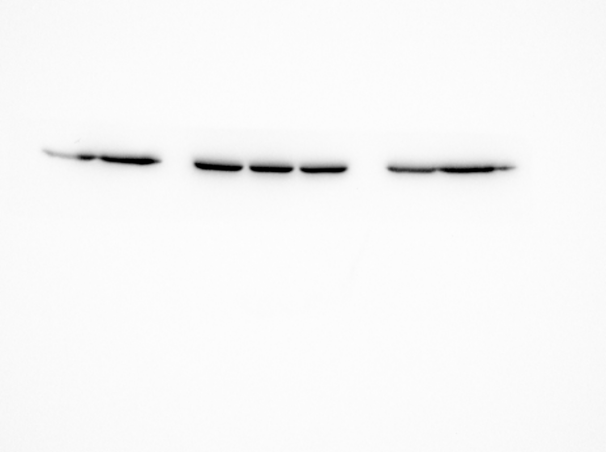


Fig 7A

SNU-739

Grouped into: control、miR-101、miR-101+UBE2D1

Left band: UBE2D1 (17kD)

Right band: β-actin (45kD)


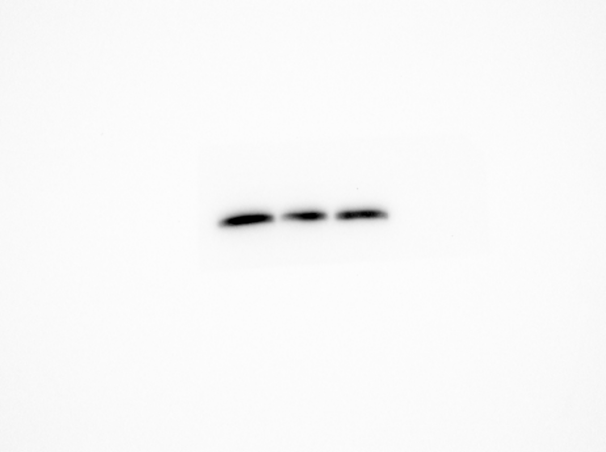

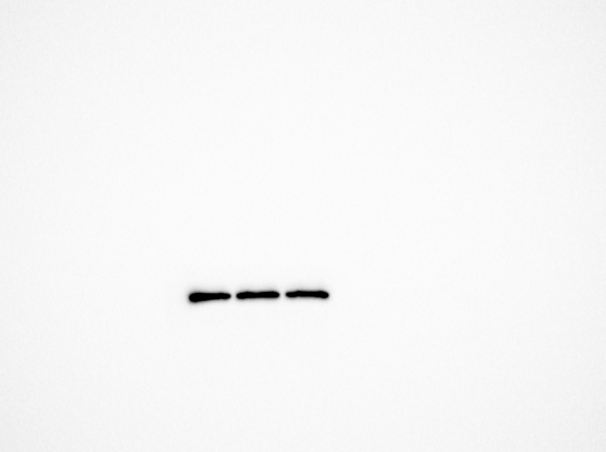


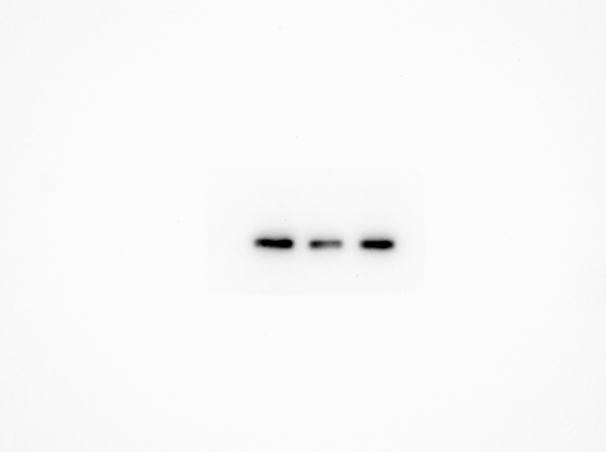

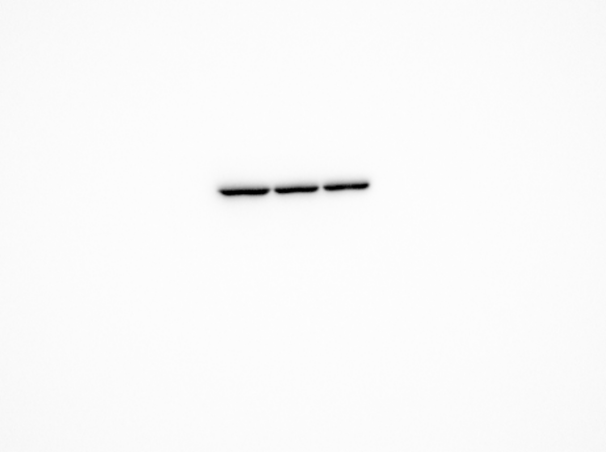


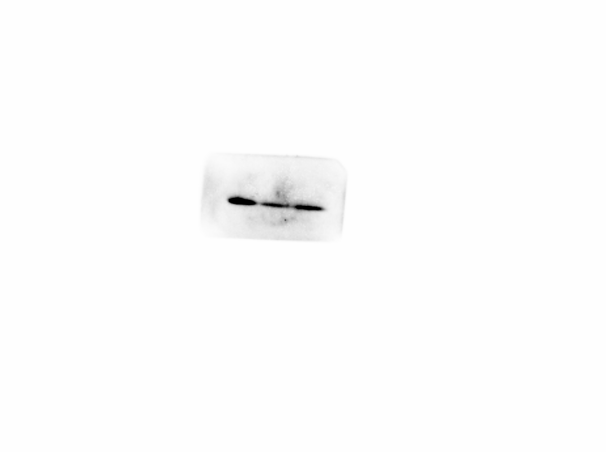

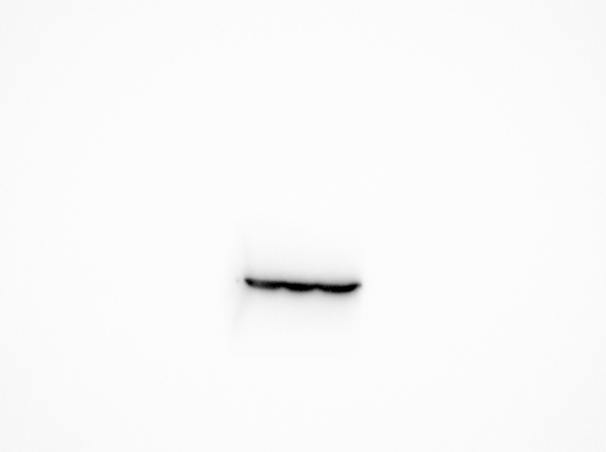


Fig 7A

HCC-LM3

Grouped into: control、miR-101、miR-101+UBE2D1

Left band: UBE2D1 (17kD)

Right band: β-actin (45kD)

Fig 7G

SNU-739

Grouped into: control、miR-101、miR-101+UBE2D1、control+5Fu、miR-101+5Fu、miR-101+UBE2D1+5Fu

Left band: cleaved caspase3 (17kD)

Right band: β-actin (45kD)

Fig 7G

HCC-LM3

Grouped into: control、miR-101、miR-101+UBE2D1、control+cDDP、miR-101+cDDP、miR-101+UBE2D1+cDDP

Left band: cleaved caspase3 (17kD)

Right band: β-actin (45kD)

Fig 7I

SNU-739

Grouped into: control、miR-101、miR-101+UBE2D1、control+5Fu、miR-101+5Fu、miR-101+UBE2D1+5Fu

Left band: γ-H2AX (15kD)

Right band: β-actin (45kD)

Fig 7I

HCC-LM3

Grouped into: control、miR-101、miR-101+UBE2D1、control+cDDP、miR-101+cDDP、miR-101+UBE2D1+cDDP

Left band: γ-H2AX (15kD)

Right band: β-actin (45kD)
