# Supplementary material for: Aberrant activation of a miR-101–UBE2D1 axis contributes to the advanced progression and chemotherapy sensitivity in human hepatocellular carcinoma
Source: Cell Death Discov. 2024 Oct 1;10:422. doi: 10.1038/s41420-024-02193-y (PMC11445525; doi:10.1038/s41420-024-02193-y)
Supplement: Supplementary file 2 — Supplemental Material [file 41420_2024_2193_MOESM2_ESM.docx]

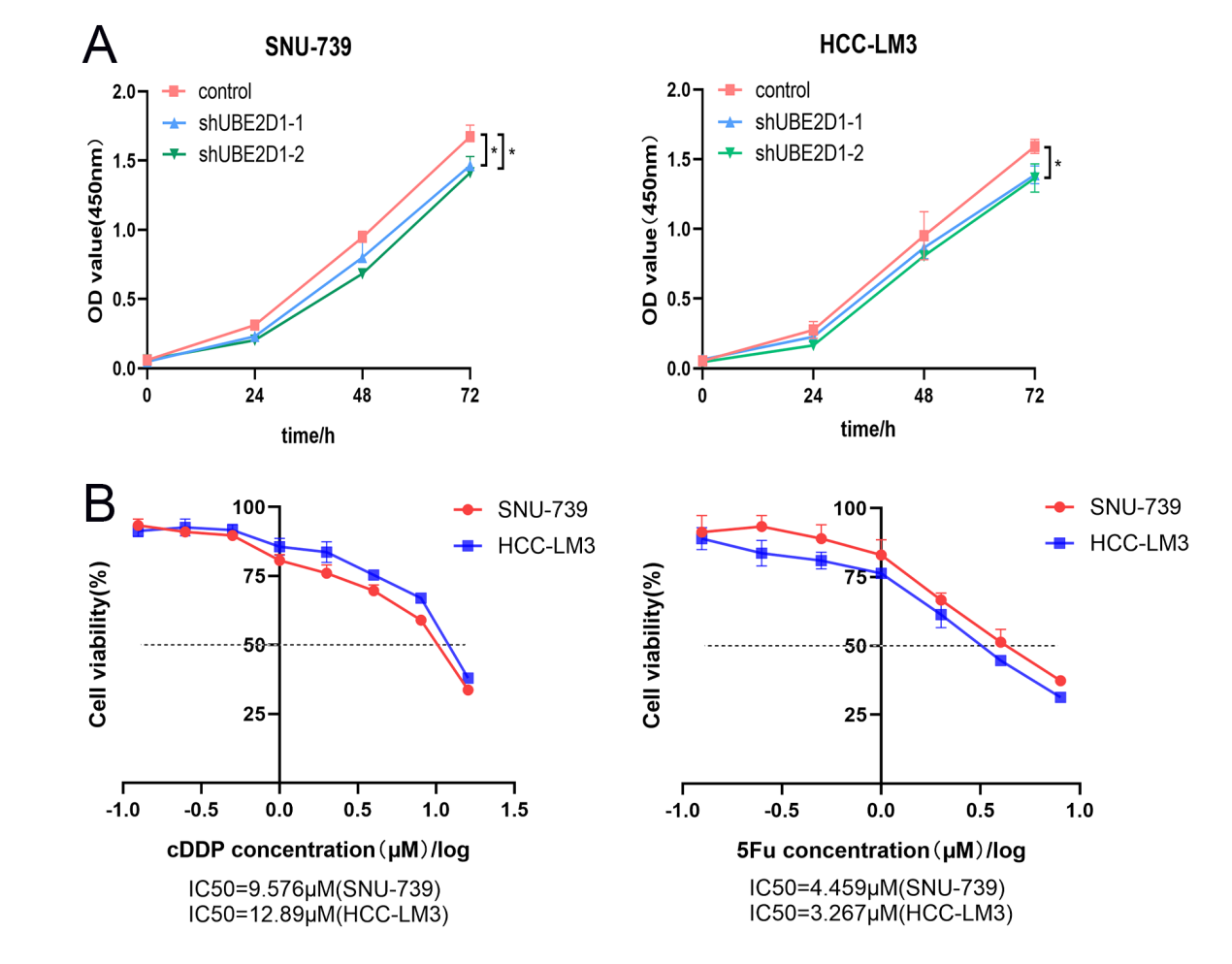


**Figure S1. (A)** The cell proliferation ability was evaluated in UBE2D1 stable-knockdown HCC cells using the CCK-8 assay. **(B)** The drug sensitivity experiments were conducted on SNU-739 and HCC-LM3 cells under different concentration gradients of cDDP and 5Fu. Statistical significance was determined by Student’s t test. ^****^*p* < 0.0001, ^*^*p* < 0.05.

Table S1. shRNA target sequences

| Target | TRC clone ID | Target sequence |
| --- | --- | --- |
| UBE2D1-1 | TRCN0000003381 | CTTCTTTCTCACTGTACATTT |
| UBE2D1-2 | TRCN0000003384 | GCGATCCACCTGCTCACTGTT |

Table S2. Cloning primers

|  |  | Sequence 5′-3′ |
| --- | --- | --- |
| PCDH-UBE2D1 | Forward primer | CCGGAATTCATGACTCCTGATAGCGCATAT |
|  | Reverse primer | TTGCGGCCGCTTACATTGCATATTTCTGAGTCC |
| pLenti6.3-UBE2D1 | Forward primer | GTACAAAAAAGCAGGCTATGACTCCTGATAGCGCATAT |
|  | Reverse primer | GTACAAGAAAGCTGGGTTTACATTGCATATTTCTGAGTCC |

Table S3. miRNA mimics and inhibitors sequences

|  |  | Sequence 5′-3′ |
| --- | --- | --- |
| NC mimics | sense | UUCUCCGAACGUGUCACGUTT |
|  | antisense | ACGUGACACGUUCGGAGAATT |
| miR-101 mimics | sense | UACAGUACUGUGAUAACUGAA |
|  | antisense | CAGUUAUCACAGUACUGUAUU |
| NC inhibitors |  | CAGUACUUUUGUGUAGUACAA |
| miR-101 inhibitors |  | UUCAGUUAUCACAGUACUGUA |
